# Essential Oils as Antimicrobial Agents—Myth or Real Alternative?

**DOI:** 10.3390/molecules24112130

**Published:** 2019-06-05

**Authors:** Katarzyna Wińska, Wanda Mączka, Jacek Łyczko, Małgorzata Grabarczyk, Anna Czubaszek, Antoni Szumny

**Affiliations:** 1 Department of Chemistry, Wrocław University of Environmental and Life Sciences, Norwida 25, 50-375 Wrocław, Poland; jacek.lyczko@upwr.edu.pl (J.Ł.); magrab@onet.pl (M.G.); antoni.szumny@upwr.edu.pl (A.S.); 2 Department of Fermentation and Cereals Technology, Wrocław University of Environmental and Life Sciences, Chełmońskiego 37/41, 51-630 Wrocław, Poland; anna.czubaszek@upwr.edu.pl

**Keywords:** essential oils, antibacterial activity, antifungal activity, lavender oil, thyme oil, peppermint oil, cajuput oil, cinnamon oil, eucalyptus oil, clove oil, sage oil, tea tree oil

## Abstract

Herbs and the essential oils derived from them have been used from the beginning of human history for different purposes. Their beneficial properties have been applied to mask unpleasant odors, attract the attention of other people, add flavor and aroma properties to prepared dishes, perfumes, and cosmetics, etc. Herbs and essential oils (EOs) have also been used in medicine because of their biological properties, such as larvicidal action, analgesic and anti-inflammatory properties, antioxidant, fungicide, and antitumor activities, and many more. Many EOs exhibit antimicrobial properties, which is extremely important in fields of science and industry, such as medicine, agriculture, or cosmetology. Among the 250 EOs which are commercially available, about a dozen possess high antimicrobial potential. According to available papers and patents, EOs seem to be a potential alternative to synthetic compounds, especially because of the resistance that has been increasingly developed by pathogenic microorganisms. In this review we summarize the latest research studies about the most-active EOs that are known and used because of their antimicrobial properties. Finally, it is noteworthy that the antimicrobial activities of EOs are not preeminent for all strains. Further investigations should, thus, focus on targeting EOs and microorganisms.

## 1. Introduction

Essential oils (EOs) are defined as volatile secondary metabolites of plants that give the plant a distinctive smell, taste, or both. EOs are produced by more than 17,500 species of plants from many angiosperm families, e.g., *Lamiaceae*, *Rutaceae*, *Myrtaceae*, *Zingiberaceae*, and *Asteraceae*, but only about 300 of them are commercialized [1]. Compounds included in the EOs are synthesized in the cytoplasm and plastids of plant cells through the pathways of malonic acid, mevalonic acid, and methyl-d-erythritol-4-phosphate (MEP). They are produced and stored in complex secretory structures, such as glands, secretory cavities, and resin conduits, and are present as drops of liquid in the leaves, stems, flowers and fruits, bark, and roots of plants. Despite containing two or three main components at a level of 20–70%, EOs are very complex mixtures of mainly terpenes, terpenoids, and phenylpropanoids. They may also contain many other compounds, such as fatty acids, oxides, and sulfur derivatives [2].

EOs are usually obtained as a result of hydrodistillation, steam distillation, dry distillation, or the mechanical cold pressing of plants. At the laboratory scale, the classical method is based on the use of the Clevenger steam distillation apparatus, discovered in 1928. Due to several disadvantages (i.e., placement of valve, fragility), this apparatus was modified by Jakub Deryng in 1951 [3] and it is widely used in Central European countries. Modifications of the simultaneous distillation-extraction (SDE) equipment were described in the manuscript of Arora et al. [4]. The effectiveness of these modifications was described in detail by Baj et al. [5]. At the laboratory scale, modern methods also include processes supported by microwaves and extraction in supercritical fluids. EOs can also be isolated using fermentation, crushing, extraction, or hydrolysis. However, depending on the chosen method, the chemical composition of the obtained EO can unfortunately be different.

Humans have used EOs for thousands of years, not only as ingredients of perfumes or as seasonings for the aromatization of food, but also in folk medicine, because of their many different biological properties, including antimicrobial properties [6]. The antimicrobial qualities are essential in managing the rapidly growing issue of drug-resistant microorganisms. In 2016, about 6 million people died globally due to infections of the upper respiratory tract, tuberculosis, or diarrheal diseases. At the same time, the number of strains of microorganisms resistant to existing antibiotics is constantly increasing. Patients with infections caused by drug-resistant bacteria are, thus, exposed to an increased risk of worse clinical results and even death. Such patients also consume more healthcare resources than patients infected with non-resistant strains of the same bacteria. According to the WHO report on drug resistance, the most serious problems include the resistance of *Klebsiella pneumoniae* to third-generation cephalosporins and carbapenem, *Escherichia coli* to third-generation cephalosporins and fluoroquinolone, *Staphylococcus aureus* to methicillin, *Streptococcus pneumoniae* to penicillin, and *Salmonella* sp. to fluoroquinolones. Among the fungal infections, the most common problem is candidiasis caused mainly by *Candida albicans* and less often by *C. glabrata* and *C. parapsilosis*, with more than 20 species of *Candida* that can cause human infection [7]. Other examples of common fungal infections are aspergillosis, histoplasmosis, and skin mycosis (commonly known as ringworm) [8]. 

In food production, inhibiting the growth of microorganisms through the use of socially acceptable preservatives is a serious problem. Society’s reluctance to use antibiotics and synthetic preservatives, such as benzoic acid, sorbic acid, lactic acid, propionic acid, acetic acid, and its derivatives, parabens or inorganic sulfites, nitrites, and nitrates, necessitates finding alternative solutions [9]. This may be an application for EOs, especially since chemical preservatives cannot eliminate several pathogenic bacteria, such as *Listeria monocytogenes*, in food products or delay the growth of spoilage microorganisms. In addition, natural products are inherently better tolerated in the human body, usually with fewer side effects [10].

## 2. Description of EOs

In the following section, the antimicrobial activity of selected EOs from above-mentioned aromatic and medicinal plants is discussed. The table of selected oils and strains of microorganisms they are active against is part of the supplementary information (Table S2). Additionally, in the supplementary information, we have included GC-MS chromatograms (Figures S1–S9) and a summary table of ingredients discussed in the study of oils (Table S1).

### 2.1. Lavender EO

#### 2.1.1. The Sources of Lavender EO

Lavender is one of the most commonly cultivated plants in the world on account of its EO properties. The main cultivation areas of lavender are in Europe, the Middle East, Asia, and North Africa [11]. Lavender belongs to the *Lamiaceae* family, formally called *Labiatea*. The genus *Lavandula* includes about 40 different species and hundreds of varieties and its hybrids. The variability of the composition between different species is described in the review article written by Aprotosoaie et al. [12]. The three species most commonly grown types are: *L. angustifolia* Mill. (narrow-leaved lavender, usually medical), *L. stoechas* (French lavender), *L. latifolia*, and their hybrids. *L. angustifolia* Mill. (formerly synonymous with *L. officinalis* L. or *L. vera* DC), is a species with the most significant industrial importance because of the EO derived from it. EO of lavender has unique biological activity, not only antimicrobial properties [13]. World trade in lavender EO is estimated at 50 million dollars [14].

#### 2.1.2. Chemical Composition of Lavender EO

Lavender EO is a colorless or pale yellow liquid with a characteristic odor. The chemical composition is well known, with detailed data specified in the European Pharmacopoeia 9, Polish Pharmacopoeia VIII, and PNISO (Polish ISO standard) 3515: 2004. Lavender EO is obtained after distillation with water vapor of fresh or dried tips of blooming plants. The main components are *R* enantiomers of linalool (20–45%) and linalyl acetate (25 to 46%). The high content of these ingredients determines the quality of the oil. The content of other ingredients should be in the following ranges: limonene (>1.0%), eucalyptol (<2.5%), camphor (>1.2%), terpin-4-ol (0.1–6.0) %), lawandulol (<0.1%), lavandulyl acetate (<0.2%), and α-terpineol (>2.0%). Due to the incalculable influence on the scent, lavender oil should not contain too much ocymen, cineole, camphor, or terpin-4-ol [15,16].

#### 2.1.3. Antimicrobial Properties of Lavender EO

Lavender EO showed antiviral activity against *Herpes simplex* virus type 1 [17]; it has shown antibacterial activity to a much greater extent. In dermatology, lavender EO can be used to treat ulcers, burns, and scarring that are difficult to cure. An extensive review of the properties of many different oils, including lavender EO, in relation to the pathogens responsible for dermatological diseases is detailed in Orchard et al. [18]. It is worth noting that the EO of *L. angustifolia* Mill. has a strong antiseptic effect against antibiotic-resistant strains, e.g., *Staphylococcus aureus* (MRSA) resistant to methicillin or vancomycin-resistant strains of *Enterococcus* sp. (VRE) [19,20]. Lavender EO is also active against the strain of *E. coli* J53 R1 resistant to piperacillin. Essential oil caused the strain’s sensitivity to this antibiotic by altering the permeability of the outer membrane, as demonstrated by decreased bioluminescence. The activity of lavender EO against 24 strains of *L. monocytogenes* was also tested (minimal inhibitory concentration (MIC) 2.5–5.0 μL/mL). In the case of *L. monocytogenes* L120 resistant to chloramphenicol, the MIC was 1.3 μL/mL [21].

The activity of lavender EO against *Shigella flexneri*, which causes dysentery, has also been demonstrated [22]. Researchers have found that antimicrobial activity of this EO is a result of the synergistic action of major and minor components of the essential oil. In turn, using commercial *L. angustifolia* Mill. EO (Pollena Aroma, Warsaw), it was shown that the non-sporulating Gram-negative bacteria from the *Enterobacteriaceae* family (MBC = 1.25 and 2.5 mg/mL) were more sensitive to essential oil than Gram-positive *S. aureus* bacteria (MBC = 5 mg/mL) [23].

Lavender EO also has antibacterial activity against clinical strains of bacteria, which were isolated from patients with respiratory tract infections [24]. It is used to treat infections of the mouth, throat, upper respiratory tract, and lungs [25]. In addition, antibacterial activity may be accompanied by an immunostimulatory effect. After inhalation, no sputum bacteria were found in the essential oil, while the symptoms and increased chemiluminescence of granulocytes were alleviated and the incidence of infections was reduced in patients with bacterial respiratory infections [19]. *Haemophilus influenzae* may be responsible for such infections and lavender EO inhibited the growth of strains that were resistant to erythromycin or amoxycycline [25].

Lavender EO can be used in chicken farming because it inhibits the growth of microorganisms (MIC % *v*/*v*), such as *S. aureus* ATCC 25923 (0.25), *S. pullorum* ATCC 13036 (0.50), *C. albicans* ATCC 10231 (0.625), *E. coli* ATCC 25922 (0.625), *S. enteritidis* ATCC 13076 (0.625), *S. aureus* MRSA/ORSA (0.625), *E. coli* [enro (−)] (1.0), *P. aeruginosa* (2.0), and *S. typhimurium* ATCC 14028 (5.0).) Additionally, when applied in a concentration of 0.4 mL/L in drinking water, it increased chicken body mass [26]. Lavender EO also showed antifungal activity because it was active against *Aureobasidium pullulans*, *Penicillium citrinum*, and *Penicillium simplicissimum* in a concentration of 87 µg/mL [27]. Lavender EO was less active than thyme and oregano against *Botrytis cinerea* and *Fusarium solani* var. *coeruleum,* [28], as well as *Aspergillus niger and Aspergillus tubingensis* isolated from grapes (minimum inhibitory doses (MID) = 0.313 μL/cm^−3^ of air) [29].

### 2.2. Thyme EO

#### 2.2.1. The Sources of Thyme EO

The genus *Thymus*, consisting of over 400 species, belongs to the *Lamiaceae* family. Thyme grows as a shrub with a stumpy, raised stem, on which small, longitudinal leaves and two-lobed pink-violet, light-violet, or white flowers in pairs glow. Oblong, brown splines are obstructions This plant has a height of 10–40 cm. EO is found in glandular hairs on leaves and flowers [15,16]. Plants of this type have been used in folk medicine for thousands of years, especially in the countries of the Mediterranean region. Antimicrobial activity is the most extensively studied in the case of *Thymus vulgaris* L. (common thyme, German thyme) [30]. The high intraspecific polymorphism of *T. vulgaris* L. in the production of terpenes is noteworthy, since 6 chemotypes of this species were found [31,32]. Pharmacopeia refers to thymol chemotype. Thyme EO (Latin *thymi aetheroleum*) is obtained by steam distillation of fresh aerial parts of *T. vulgaris* L.; *T. zygis* Loefl (Spanish thyme, white thyme), or mixtures of both species. The highest level of EO production usually occurs during the flowering period of the plant [30].

#### 2.2.2. Chemical Composition of Thyme EO

The composition of thyme EO is well known and described. The main components of thyme EO are thymol (36–55%) and *p*-cymene (15–28%). It should be noted that the thymol was first isolated in 1719 by Neuman. The compound is characterized by strong bactericidal, fungal, and anti-parasitic properties, with relatively low toxicity to humans and animals. The percentage of other compounds should be in the following ranges: γ-terpinene (5–10%), linalool (4–6.5%), carvacrol (1–4%), β-myrcene (1–3%), terpin-4-ol (0.2–4.0%) [33]. In addition, there are also monoterpene hydrocarbons, such as α-tujen, α-pinene, α-terpinen, camphor myrcen, as well as oxygen derivatives of monoterpenes, 1.8-cineol, linalool, terpin-4-ol, and γ-terpinol, camphor and borneol. Sesquiterpene hydrocarbons have also been identified, which include α-humulene, α-kopene, kubeben, α-gurjunen, germacren D, α- and γ-muurolen, and γ and δ-cadinene [15,16].

#### 2.2.3. Antimicrobial Properties of Thyme EO

Thyme EO showed antiviral activity, as it was active against *Herpes simplex* (HSV1, DNA virus) with IC_50_ values of 11 µg/mL [34]. This EO also displayed 100% inhibitory activity in the liquid phase at 3.1 µL/mL concentration against influenza virus A1/Denver/1/57 (H1N1) with 30 min exposure [35]. Nevertheless, the most important effect of thyme EO relates to bacteria. Particularly high bacteriostatic activity against the majority of Gram-positive and Gram-negative bacteria is demonstrated by the thymol chemotype of *T. vulgaris* L. EO, which has been described in many reviews [30,36,37,38,39]. This activity is due to the high content of thymol and carvacrol, which due to the acidic nature of the hydroxyl group, can form hydrogen bonds with the active site of many enzymes. In 2017, Schött [40] described the activity of thyme EOs against *S. mutans*. This bacteria is responsible for the development of dental caries but may also cause infective endocarditis. The EO from *T. zygis* was more active than EO from *T. vulgaris*. Only thymol chemotype was active.

The activity of thyme EO against strains responsible for acute bacterial pharyngitis and throat inflammation was also examined. This infection is caused by β-haemolytic *Streptococci* strains, e.g., *S. pyogenes*. *T. vulgaris* EO was active against the *S. pyogenes* strains isolated from the throat of patients [41]. The activity of thyme EO and carvacrol was examined against *Malassezia furfur*—an opportunistic yeast that can develop on the face, forearms, and scalp, especially during adolescence. The MIC was in the range of 450–900 μg/mL [42].

Thyme EO was active against *S. aureus* ATCC 25923 and *K. pneumoniae* ATCC 13882. It also inhibited the growth of *Brachyspira hyodysenteriae*, which causes swine dysentery [30].

On the other hand, thyme EO rich in γ-terpinene (68.415%) and *p*-thymol (24.721%) completely inhibited the growth of *Fusarium graminearum* Fg 06–17 at a concentration of 0.06% [43]. However, again the antifungal activity of thyme EO is mainly attributed to the presence of thymol, which may, due to its low polarity, interfere with the lipid bilayer of the cell membrane, changing its fluidity and permeability. Other mechanisms of action are based on cell wall damage and changes in fungal morphology. Thyme EO inhibited aflatoxin production, caused the cytoplasmic spill of *A. flavus*, and was responsible for degenerative changes in its hyphae, which even led to complete disappearance of conidia [44]. It also influenced the morphology and growth of *A. niger* [45]. Thyme EO showed a synergistic effect in combination with cinnamon and rosemary EOs on *Botrytis cinerea* ATCC12481 and *Penicillium expansum*. The mean lesion diameter of the pears treated with the cinnamon-rosemary-thyme EOs combination (78, 1250, 39 μg/mL, respectively) was 6 mm and 8 mm against *B. cinerea* and *P. expansum*, respectively, after 10 days at 25 °C [46].

### 2.3. Peppermint EO

#### 2.3.1. The Sources of Peppermint EO

The genus Mentha is part of the family of Lamiaceae (*Lamiaceae*) and includes about 30 species grown in temperate climate zones around the world, especially in Europe, North America, North Africa (Syria, Ethiopia), and northern parts of Iran. Due to the high variability of the species and great ease of crossing species, the chemical composition of the EOs obtained from them is very diverse [2]. One of the species—peppermint—is a natural hybrid of two species: *Mentha spicata* L. and *Mentha aquatic* L. Peppermint is a perennial with a height of 30–90 cm with numerous underground and aboveground runners and a purple stem. The leaves are oblong and dark green. Purple flowers are collected in capitate inflorescences. This plant requires fertile and permeable soil. EO from peppermint is obtained after distillation of dried leaves with water vapor. As a result of this process, a light yellow or greenish liquid with an intense mint aroma is obtained [15,16,47]. 

#### 2.3.2. Chemical Composition of Peppermint EO

Approximately 300 compounds have been identified in the EO. The main components are menthol (30–55%) and mentone (14–32%). Menthol occurs mainly as the isomer with the (1*R*, 3*R*, 4*S*) configuration (20–60%), while the main isomer of mentone is (1*R*, 4*S*) (5–35%). The Polish Pharmacopoeia VIII defines the content of other ingredients as follows: cineol (3.5–14%), menthyl acetate (2.8–10%), isomenton (1.5–10%), menthofuran (1.0–9.0%), limonene (1.0–5.0%), pulegone (<4.0%), and carvone (<1.0%). The quantitative composition of EO depends on many factors, such as the growing conditions, and the date of harvesting. Therefore, the date of the congregation should be chosen to ensure that it contains as much menthol as possible [15,16,33].

#### 2.3.3. Antimicrobial Properties of Peppermint EO

The broad spectrum of biological activity of plants of the genus *Mentha* was discussed recently in a review article [2]. Peppermint essential oil exhibited high levels of virucidal activity against HSV-1 and HSV-2 in viral suspension tests. Both kinds of viruses were significantly inhibited when *Herpes simplex* virus was pre-treated with the EO prior to adsorption. Peppermint EO affected the virus before adsorption, but not after penetration into the host cell [48].

Peppermint EO has a weak antibacterial activity, hence it is usually included in complex preparations. Its widespread use is due more to a pleasant mint flavor and the feeling of coolness than to its antimicrobial properties. However, it is believed that the higher menthol content in peppermint EO has more antimicrobial activity [18]. In the diffusion test, peppermint EO (20 μL) inhibited the growth of bacterial strains, such as *E. coli* WDCM 00013, *L. monocytogenes* WDCM 00020, *P. aeruginosa* WDCM 00024, *S. enterica* WDCM 00030, and *S. aureus* WDCM 00032. The inhibition zone was from 12 mm for *P. aeruginosa* up to 37.66 mm for. *S. aureus* [49].

Recently, the effect of peppermint EO on the development of yeast has been investigated. These microorganisms may be responsible for the spoiling of cashew, guava, mango, and pineapple juices. In preliminary studies, the MIC was 1.875 μL/mL against *C. albicans*, *C. tropicalis*, *Pichia anomala*, and *S. cerevisiae*. However, when used in mango and pineapple juices, even at higher concentrations of EO (7.5 μL/mL), no significant reduction in yeast was observed. In the case of *S. cerevisiae*, the addition of 1.875 μL/mL of mint EO to the cashew and guava juices strongly weakened the membrane permeability, membrane potential, and activity of the efflux pump in the yeast cells. It is true that mint EO did not affect the appearance, smell, and viscosity of fruit juices, but negatively influenced their taste [50]. In turn, Benzaid et al. [7] determined that mint EO in the volatile form inhibits the development of *C. albicans* comparable to amphotericin-B, influencing the expression of various genes, such as secreted aspartyl proteinases (SAP 1, 2, 3, 9, 10), and being associated with the process of adhesion of hyphal wall protein 1 (HWP1).

It is worth noting that although the mint EO alone has a weak antibacterial activity, it may have a synergistic effect with other EOs or substances. For example, it increased the activity of *Pongamia pinnata* EO and additionally increased over 30 times the sensitivity of bacteria to gentamicin, *E. coli* pMG309 harboring and plasmid encoding β-lactamase, KPC-3 on meropenem, and caused a strong anti-candidal effect with azole antibiotics, such as Fluconazole and Ketoconazole, and a weaker synergistic effect with Clotrimazole and Itraconazole. The research was carried out using *Candida* strains isolated from patients affected by skin diseases and urine samples [51].

One of the popular preparations used to treat headaches, colds, coughs, mild spinal gastrointestinal complaints, and to relieve local muscle pain is Olbas^®^ Tropfen (Olbas). It contains EOs such as peppermint EO (5.3g), eucalyptus (2.1g), cajuput, and a smaller amount of juniper EO (0.3g). Olbas^®^ has shown antibacterial activity against many strains, including methilicin-resistant *S. aureus* (MRSA) and vancomycin-resistant *Enterococcus*. Hence, Olbas^®^ could be used to treat uncomplicated skin and respiratory infections [52]. This activity may result from the high content of monoterpenes, especially menthol, which due to their hydrophobicity, affect the fluidity and permeability of the cell membrane. Monoterpenes also affect the conformation of proteins embedded in the membrane, thus inhibiting the process of cellular respiration and disrupting the transport of ions through cell membranes, which can lead to cell death [53,54]. 

The possibility of using peppermint EO introduced into a chitosan nanogel was investigated in the protection of plaque against *S. mutans* causing caries. The maximum release of peppermint EO from the nanogel was about 50% after 360 h in an aqueous-alcoholic solvent at ambient temperature. The adhesion of bacterial cells was highly sensitive to nanoformulation of mint EO, as compared to the unloaded chitosan nanogel. Inhibition of biosynthesis of *S. mutans* occurred at a concentration of 50 μg/mL, compared to 400 μg/mL for a nanogel without EO. In addition, it was found that chitosan nanogel containing mint EO inhibited the activity of glycosyltransferase genes (*gtf*B, *gtf*C, and *gtf*D) involved in the formation of extracellular polymers [55]. Moreover, peppermint EO also inhibited the growth of fungi strains, such as *Alternaria alternata*, *Aspergillus flavus*, *Aspergillus niger*, *Colletotrichum gloeosporioides*, *Fusarium solani*, and *Macrophomina phaseol* [56].

### 2.4. Cajuput EO

#### 2.4.1. Source

Cajuput oil is obtained from leaves and small branches of the cajuput tree (*Melaleuca leucadendron* L.; syn of *M. cajuputi* Powell, syn of *M. minor* Smith), which belongs to the *Myrtaceae* family and is native to Southeast Asia and North East Australia [57,58]. The cajuput tree reaches a height of 25 m, and has a characteristic white and twisted trunk, similar to birch trees. Furthermore, the bark of the cajuput tree has a thick flaky texture that detaches itself from the trunk and hangs downwards [59]. The branches are arranged irregularly, covered with white bark, and in the case of young branches, slightly silvery. The leaves are thick, lanceolate, about 10 cm long, and 1.5 cm wide. Oil reservoirs are visible on the leaf surface, making the leaves highly aromatic. Their smell is associated with camphor, rosemary, and cardamom. The small, white, individual flowers are collected in spiky inflorescences. Flowers bloom in early summer or spring and developed fruits are green-brown sacks [60].

The cajuput EO is obtained both from wild plants and those cultivated on plantations. It is obtained as a result of distillation with steam from fresh twigs. The cajuput EO content ranges from 1.5–3.0% [61]. The cajuput EO is yellow-green with an intense, herbal scent reminiscent of eucalyptus EO.

#### 2.4.2. Chemical Composition of Cajuput EO

One of the most characteristic components of cajuput EO is 1,8-cineole (eucalyptol), which has a camphor-medical smell. Its content varies from 15–60% [62]. In addition to eucalyptol, the cajuput EO also contains terpenes, such as γ-terpinene, limonene, *p*-cymene, terpinolene, α-pinene, β-pinene, β-caryophyllene, α-humulene, aromadendren, α-selinene, and β-selinene. Moreover, the cajuput EO also contains terpenoids, such as α-terpineol, linalool, terpinen-4-ol, cajeputol, spathulenol, globulol, viridiflorol, and cariophilene oxide. Nevertheless, the main constituents of cajuput EO differ according to the origin of the EO. In Indonesia, EO extracted from *M. cajuputi* subsp. *cajuputi* is characterized by a high content of 1,8-cineneol. In Papua New Guinea, there are two chemotypes, *M. cajuputi* subsp. *platyphylla*, which differ in composition. Chemotype I contains significant amounts (2-80%) of 1-acetyl-4-methoxy-3,5,5-trimethylcyclohex-3-en-2,5-dione, while the main components of chemotype II are α-pinene, 1.8 -cineole, γ-terpinene, β-cymene, and β-caryophyllene. Furthermore, cajuput EO from Cuba contains mainly viridiflorol (28.2%) and 1.8-cineole (21.3%). In turn, the EO from South India contains (*E*)-nerolidol (76.58–90.85%), β-caryophyllene (1.52–4.49%), viridiflorol (0.19–2.79%), (*E*)-β-farnesene (≤0.10–2.67%), and α-humulene (0.22–1.03%) [5], while cajuput EO from North India contains 1.8-cineole (19.9%), β-eudesmol (15.8%), α-eudesmol (11.3%), viridifloral (8.9%), and guaiol (9.0%) [62].

#### 2.4.3. Antimicrobial Activity of Cajuput EO

Cajuput oil has been used in medicine since the eighteenth century as an antiseptic agent. The most active ingredients are 1,8-cineole, linalool, and terpinen-4-ol. Its effect is comparable to that of tea tree oil [63]. At a concentration of 0.2–0.4%, cajuput oil inhibits the growth of Gram-positive bacteria: *Bacillus cereus, Bacillus subtilis, Corynebacterium diphtheriae, Corynebacterium minutissimus, Enterococcus faecium, Listeria monocytogenes* [56], *Micrococcus luteus, Staphylococcus aureus, S. capitis, S. epidermidis, S. faecalis, Klebsiella* spp. and *Staphylococcus aureus* [52]. A higher concentration of 0.4–0.6% inhibits Gram-negative bacteria, such as *Alcaligenes faecalis, Enterobacter cloacae, Escherichia coli,* and *Proteus vulgaris,* yeast such as *Candida albicans, C. vaginalis,* and *C. glabrata*, and mold such as *Aspergillus niger* and *Penicillium notatum* [58,64,65].

### 2.5. Cinnamon EO

#### 2.5.1. Source

Cinnamon is a natural plant that grows in Sri Lanka and tropical Asian countries. It is an important spice plant widely used throughout the world. Over 250 species of the genus *Cinnamomum* are known [66].

Cinnamon oil, known for its healing properties, is a product of the distillation with water vapor of three raw materials: (i) leaves and young twigs of Chinese cinnamon (*Cinnamomoum cassia* [syn *aromaticum*] Blume); (ii) Ceylon cinnamon leaves (*Cinnamomum zeylanicum* Blume [syn *C. verum*]; and (iii) Ceylon cinnabar bark [33].

These types of cinnamon vary in appearance, taste, and place of cultivation. *C. cassia* is grown mainly in China and is characterized by a dark reddish-brown color and a sharp and bitter taste, while *C. zeylanicum* grown in Sri Lanka is characterized by a bright to medium-dark reddish-brown color and sweet taste [67]. Moreover, these are differences in the classification regarding raw material used in medicine. In India and Sri Lanka, *C. cassia* is considered a substitute for *C. zeylanicum*. In turn, Chinese, Korean, Taiwanese, and Japanese pharmacopoeias allow usage of only the first one [68].

#### 2.5.2. Chemical Composition of Cinnamon EO

The main components of cinnamon EO are *trans*-cinnamaldehyde, *o*-methoxy-cinnamaldehyde, cinnamyl aldehyde, benzaldehyde, phenylethanol, borneol, eugenol, coumarin, and cinnamic acid [69,70]. Depending on the raw material (bark or cinnamon leaves) from which the EO was obtained, the ratio of the two main components—eugenol and cinnamaldehyde—significantly differs. For EO obtained from cinnamon bark, it is characterized by a low amount of eugenol (5–10%) and abundance of cinnamyl aldehyde (65–80%), while EO obtained from cinnamon leaves is rich in eugenol (10–95%) and poor in cinnamyl aldehyde (1–5%) [71].

#### 2.5.3. Antimicrobial Activity of Cinnamon EO

In numerous studies of antimicrobial activity, cinnamon EOs of various origins are used. EO of *Cinnamomum zeylanicum* displayed 100% inhibitory activity at 3.1 µL/mL concentration against influenza virus A1/Denver/1/57 (H1N1) with 30 min exposure. Eugenol, the major component of *Cinnamomum zeylanicum* EO, possessed the most potent anti-influenza activity in both liquid and vapour phases [35]. Brochot et al. investigated the antiviral activity of a blend that was composed of equal parts (3.52% each) of *Eucalyptus globulus* CT cineol (leaf) and *Cinnamomum zeylanicum* CT cinnamaldehyde (bark), 3.00% *Rosmarinus officinalis* CT cineol (leaf), 1.04% *Daucus carota* CT carotol (seed), and 88.90% *Camelina sativa* oil (seed). This blend significantly reduced viral units of H1N1 and HSV1. For both viruses, a reduction greater than 99% was observed with 1% blend and 60-min contact time [71]. Intorasoot et al. [72] and Feng et al. [73] carried out studies involving EO from the bark of *C. zeylanicum*, which is rich in cinnamic aldehyde. They showed that the EO has a better effect compared to other EOs used (including clove EO, lemongrass, tea tree, ginger, basil), with very good activity against *S. aureus, E. coli, A. baumannii,* and *P. aeruginosa* [74], and even better activity than the antibiotic against *B. burgdorferi* [73]. Cinnamon EO can cause destruction of bacterial cell membranes [74]. Furthermore, the antimicrobial activity of cinnamaldehyde against *S. aureus* has been previously confirmed by Ferro [75]. In a study carried out by Aumeerudda-Elalfi et al. [76], *C. zeylanicum* EO from leaves was used. The experiment indicated better antimicrobial activity than antibiotics (ampicillin, chloramphenicol, tetracycline) against *E. faecalis, E. coli, P. auerginosa,* and *S. aureus*. However, it did not show better antimicrobial activity than other EOs examined during the study.

Cinnamon oil obtained from the leaves and bark of *C. zeylanicum* inhibited the growth of *Salmonella typhimurium* and *Listeria monocytogenes* (MIC = 0.5% (*v*/*v*)), which constitute a serious public health problem. *Listeria* is responsible for about 1600 cases of food poisoning in the US and 260 deaths annually, while *Salmonella* caused about 19,000 hospitalizations and 380 deaths annually in the US. Both *Salmonella* and *Listeria* can contaminate food, such as fruits, vegetables, seafood, and dairy products [6]. Furthermore, Brnawi et al. [6] investigated whether the cinnamon EO would inhibit the growth of bacteria growing on celery stored at 4 °C for 7 days. The EO at a concentration of 0.5% significantly reduced (*p* < 0.05) the growth of *S. typhimurium* (4-week-old chicken heart and liver) and *L. monocytogenes* (V7 serotype 1/2a, isolated from raw milk).

Cinnamon EO has recently been used to modify the zein film used for food packaging, which contains an additional 4% chitosan nanoparticles (CNP). The results showed that the combination of EO with nanoparticles not only gave antimicrobial properties by inhibiting the growth of *Escherichia coli* (PTCC 1163) and *Staphylococcus aureus* PTTC 25923, but also improved the tensile strength and decreased the elongation of the composite zein film [77].

A cinnamon-rich bark EO from *Cinnamomum cassia* (L.) J. Presl, rich in cinnamic aldehyde, was used to prepare a herbal toothpaste (91.8%), which inhibited the growth of *Streptococcus mutans*, which causes the development of dental caries. The toothpaste Jack N’ Jill, which initially showed no antibacterial effect, showed a 39 mm inhibition zone of *S. mutans* ATCC 35668 after adding cinnamon EO. The results of this study indicate that herbal toothpaste shows statistically higher antimicrobial activity against *S. mutans* ATCC 35668 (*p* < 0.05) after adding EOs than their initial forms without EO [78].

In another study carried out by Oliveira et al. [79], it was pointed out that the cinnamon leaf EO counteracts the development of *Candida* yeast, which can settle in the mouth. Ultimately, the antimicrobial activity of cinnamon EO against *S. aureus*, *E. coli*, and *C. albicans* was confirmed by Herman’s research [80], in which the author does not indicate the raw material from which the EO was obtained. Similarly, without giving the raw material from which the EO was obtained, Sumalan et al. [81] reported activity indicating the inhibition of the growth of filamentous fungi of the genera *Aspergillus, Cladosporium,* and *Fusarium*. Overall, the antimicrobial activity of cinnamon EO is mainly associated with the content of cinnamaldehyde, for which the MIC was in the range of 0.5 to 1000 μg/mL in relation to 20 strains of *P. aeruginosa* [66].

### 2.6. Clove EO

#### 2.6.1. Source

Clove EO is obtained from spicy clove (*Eugenia caryophyllata* Thunb, son *E. aromatica* Baill., *E. caryophyllus* L.; *Syzygium aromaticum* Merr. Et Perry). The genus *Eugenia* (*Syzygium*) belongs to the Myrtaceae family. The homeland of cloves is islands in Indonesia—the Moluckie islands. Nowadays, cloves are it is grown on large plantations in many regions, mainly on the islands of Zanzibar, Pemba, Penang, Amboine, Java, Ceylon, Madagascar, Réunion, and in Africa and South America [82]. Cloves are 15 m high trees with a dense pyramidal crown. Leaves are placed opposite each other and have an elliptic-lanceolate shape. They are leathery, dark green, and shiny on top with translucent oil reservoirs. The flowers are concentrated for 5–20 in baldachogrona. and consist of four-colored red chalices, a four-petal pink crown, and many stamens and pistils. The fruit is a dark red single- or double-edged berry. Nevertheless, the raw material from which the EO is obtained is undeveloped flower buds of *Eugenia caryophyllata* Thunb, which are subjected to steam distillation for 9–24 h after crushing. The EO yield is high and amounts to 15–20%. The EO itself is heavier than water, and colorless or light yellow. It has a durable, intense, spicy, sweet fragrance [83].

#### 2.6.2. Chemical Composition of Clove EO

Clove EO was already obtainable in the fifteenth century, and in the nineteenth century, eugenol was identified in it. At present, about 100 compounds that are components of this EO have been identified. The dominant component of clove EO is eugenol, the content of which varies from 30–95%, with the lowest content of this compound (28%) in EO isolated from growing leaves [84]. There are also eugenol acetate, β-caryophyllene, α-ilangene, δ-cadinene, as well as compounds with an aromatic structure, i.e., methyl eugenol, anetol, chavikol, vanillin, benzyl alcohol, cinnamic aldehyde, benzyl salicylate, and calamenene.

The chemical composition of the oils depends on the origin of the oil and the degree of development of the leaves or pitch. Leaf oil differs from the bud oil, with low content of eugenyl acetate.

The Polish Pharmacopoeia defines the content of the following components in the essential oil: β-caryophyllene (5–14%), eugenol (75–88%), and eugenol acetate (4–15%) [15,16,33]

#### 2.6.3. Antimicrobial Activity of Clove EO

The in vitro antiviral activity of eugenol—the main component of clove EO—has been tested against HSV-1 and HSV-2 viruses [85]. The replication of these viruses was inhibited with IC_50_ values of 25.6 μg/mL and 16.2 μg/mL against HSV-1 and HSV-2, respectively. Additional investigations revealed synergistic interactions with a combination of eugenol and acyclovir, a known antiviral drug. Studies have shown that application of eugenol delayed the development of herpes virus-induced keratitis in a mouse model [34]. Eugenol was evaluated for its anti-HSV properties on the plaque reduction assay. Only two isolates were inhibited by eugenol, but the inhibition against these isolates was greater than for the extract obtained from the flower buds of *E. caryophyllata* [86].

The antimicrobial activity of clove EO was compared with cinnamon EO, oregano, and bay leaves against medically important bacterial strains, such as *Bacillus cereus, Escherichia coli, Listeria innocua,* and *Salmonella typhimurium*. The diffusion test showed that clove EO was the most active, with MIC ranging from 1.25% (*v*/*v*) (*B. cereus*) to 2.50% (*v*/*v*) (*S. typhimurium* and *E. coli*). In addition, a synergistic effect of the clove EO with 1.8% NaCl (g/*v*) was observed, which can be used to improve the organoleptic properties of food [10,87]. Moreover, cloves are commonly used in the food industry as a natural additive or antiseptic to extend the shelf life due to the effective antibacterial action against certain food-borne pathogens [10].

Condo et al. determined that clove EO was active against pathogenic strains, such as *Aeromonas hydrophila* ATCC 7966 (IZr of 8 mm), *Candida albicans* ATCC 10231 (IZr of 7 mm), and *Proteus mirabilis* ATCC 10005 (IZr of 6 mm), while it showed a weaker effect against *Escherichia coli* ATCC 8739, *Klebsiella pneumoniae* ATCC 13883, *Pseudomonas aeruginosa* ATCC 27853, *Staphylococcus aureus* ATCC 6538, and *Staphylococcus epidermidis* ATCC 14990 [88]. Clove EO also inhibits the development of microorganisms. such as *Bacilus subtilis, Morganella morganii* [10] *Mycobacterium phlei, Aspergillus niger*, and *Penicillum christopherum* [84].

Due to the similarity of the composition of EO, tests of the antimicrobial activity of clove EO and cinnamon leaf EO are often carried out in parallel. Both EOs showed strong bacteriostatic activity at concentrations of 0.03–0.05% and bactericidal activity at concentrations of 0.04–0.1% in relation to *S. aureus, L. monocytogenes, S. enteritidis, C. jejuni,* and *E. coli*. The activity of both EOs was also compared in the case of *Yersinia enterocolitica* and 5 strains of *Salmonella enteritidis* isolated in the hospital. Clove EO was slightly weaker than cinnamon EO in relation to strains of bacteria attacking the human respiratory system, such as *K. pneumoniae, Haemophilus influenzae*, *Streptococcus agalactiae*, and *S. pyogenes*. The antimicrobial properties of clove EO with cardamon EO, cinnamon EO, and 10% phenol solution were also compared. The research was carried out using the diffusion method with 9 strains of Gram-positive bacteria, 4 Gram-negative bacteria, and 7 strains of molds. Clove EO was the most active. Schmidt et al. [89] conducted a study in which eugenol-rich clove EO was active against 38 strains of *Candida albicans* isolated from the oropharynx, damaged skin, and vagina. Interestingly, eugenol itself showed weaker activity than the clove EO isolated from leaves.

It has been proven that compounds forming clove EO can destroy the wall and cellular membrane of microorganisms, pass through cytoplasmic membranes or enter cells, and then inhibit the proper synthesis of DNA and proteins. In the case of *L. monocytogenes* treated with clove EO, the DNA content decreased from 15.004 μg/mL to 5,587 μg/mL, thus by 62.76%. In addition, the bioluminescence assay showed 81.7% reduction in intracellular ATPase, which means that clove oil can damage the cell membrane and cause ATP outflow from the cell. This oil also inhibits the action of β-galactosidase, which by hydrolyzing lactose to galactose and glucose determines the use of this sugar as a source of carbon and energy. It also inhibits bacterial respiratory metabolism by affecting the tricarboxylic acid cycle pathway [90]. In another study, it was found that eugenol, the main component of the EO, can inhibit the production of α-amylase, protease, and subtilisin by *Bacillus subtilis*, with the ability to destroy the cell wall and cause lysis of cells [91].

### 2.7. Eucalyptus EO

#### 2.7.1. Source

Eucalyptus oil is obtained from the leaves of eucalyptus globose (*Eucalyptus globulus* Labill) [33], which belongs to the *Myrtaceae* family and is the dominant plant in the forests of Australia [92]. Eucalypti are evergreen trees or shrubs that reach a height of up to 100 m. Most species drop their barks every year. Leaves of *E. globulus*, from which oil is obtained, should be greyish-green, up to 25 cm long and up to 5 cm wide, thick, and sickle-shaped. The flowers are usually white, but in some species yellow, pink, or red.

#### 2.7.2. Chemical Composition of Eucalyptus EO

The main components of eucalyptus EO are 1,8-cineol (eucalyptol), limonene, α-pinene, γ-terpinene, and α-terpineol [93]. Further, 1,8-cineol and α-pinene are also present in a similar amount in *E. maideni, E. astrengens, E. cinerea, E. leucoxylon, E. lehmani, E. sideroxylon*, and *E. bicostata* [94], which according to the Polish Pharmacopoeia [33], are not sources of eucalyptus EO, despite the fact that the content of 1,8-cineol varies from 49.07 to 83.59%, and α-pinene varies from 1.27 to 5.02% in those plants. The European and British Pharmacopoeia indicate that eucalyptus EO used for medicinal purposes must contain at least 70% 1,8-cineole [95].

#### 2.7.3. Antimicrobial Activity of Eucalyptus EO

Eucalyptus EO has antimicrobial activity against viruses, bacteria, yeasts, and filamentous fungi. Eucalyptus EO, rich in 1,8-cineole (88%), was active against HSV-1 in vitro. EO was able to suppress viral multiplication by >96%. This EO exhibits an anti-HSV1 activity by directly inactivating free-virus particles and might also interfere with virion envelope structures required for entry into host cells [34]. In turn, the antiviral activity of *Eucalyptus* species grown in Tunisia against Coxsakievirus B3 Nancy strain was studied using Confluent Vero cell cultures that were treated with non-cytotoxic concentrations of the EOs, both during and after viral infections; activity depended on *Eucalyptus* species. EOs from *E. sideroxylon*, *E. lehmannii*, *E. leucoxylon*, and *E. odorata* had no antiviral activity, while those from *E. bicostata* (IC_50_ = 0.7–4.8 mg/mL), *E. astringens*, and *E. maidennii* (IC_50_ = 136.5–233.5 mg/mL) were the most active against viral infections [96,97]. *Eucalyptus globulus* EO was mildly active against mumps virus isolated from clinical specimens of patients with respiratory tract infection. The antiviral activity was assessed by plaque reduction assay [98]. This EO also displayed 100% inhibitory activity at 3.1 µL/mL concentration against influenza virus A1/Denver/1/57 (H1N1) after 10 min exposure [35].

Antibacterial activity has been determined to a much greater extent. It is active, for example, against bacteria such as *P. gingivalis* and *S. mutant*, which cause periodontitis and other dental ailments. Therefore, its main component 1,8-cineneol has been used in products for oral hygiene, such as Listerine Cleanmint (Pfizer), Listerine Freshmint (Pgizer), and Walgreens Fresh Breath Antiseptic Mouth Rinse (Walgreen Co.) [99]. The EO was also active against *E. tarda, V. ichthoenteri, V. harveyi, P. damselae, S. iniae, S. parauberis,* and *L. garviae*, considered to be pathogens of fish [100]. Furthermore, it exhibits toxic effects against *S. aureus* and *E. coli* [101]. Eucalyptus EO is more active against Gram-negative than Gram-positive bacteria, which is attributed to the presence of components such as 1.8-cineole, *p*-cymene, *cis*-geraniol, and terpinolene, which can increase the permeability of the cytoplasmic membrane through functional impairment. The most sensitive bacterium is *Acinetobacter baumannii* [99].

In the case of filamentous fungi, Martins et al. [102] have shown that eucalyptus EO is toxic for *M. hiemalis* Wehmer, *Alternaria alternata* (Fr.) Keissl, *Penicillium glabrum* (Wehmer), and *Fusarium roseum* (Link). In turn, Tyagi et al. [96] had shown activity of the oil in relation to yeasts *S. cerevisiae, Z. bailli, A. pullulans, C. diversa, P. fermentans, P. kluyveri, P. anomala*, and *H. polymorpha*, and indicated the possibility of using eucalyptus EO in the food industry as a preservative in beverages. Eucalyptus EO toxicity studies performed in vitro and in animals have shown that it is low in toxicity. In addition, studies conducted with the participation of human volunteers indicated its low allergenicity [99].

### 2.8. Sage EO

#### 2.8.1. Source

The Salvia genus is probably the largest genus in the *Lamiaceae* family, consisting of almost 1000 species [9]. Salvia was used in medicine as early as in ancient times and the Latin name of the plant comes from the words *salvare*, to cure, and *officina*, a pharmacy. Sage EO is obtained from flowering plants or only sage leaves (*Salvia officinalis* L. syn *S. graniflor* Ten.). Sage comes from the Middle East, but grows throughout Europe, as well as in Asia Minor and North America [103]. It is a small shrub (20–70 cm) with silvery-grey hairy leaves. White or purple flowers appear in May or June. Salvia prefers environments with good sunlight and fertile soil that is rich in calcium [15,16]. Sage EO is extracted by distillation with superheated steam from partly dried raw material. The duration of the process is about 1 h and the efficiency varies between 1 and 2%.

#### 2.8.2. Chemical Composition of Sage EO

Over 120 constituents were found in sage EO [103]. Its main components are ketones and monoterpne hydrocarbons, such as borneol, camphor, 1.8-cineole, camphene, limonene, α-pinene, β-pinene, α-thujone, β-thujone, α-humulene sesquiterpene derivatives, and β-caryophyllene. In the sage EO, α-humulene [104,105], ledene [90], viridiflorol [106,107], manool [108], and sclareol [109] were also found The diversity of constituents requires classification into divisions depending on the chemotype. Tucker and Maciarello [110] described five groups based on four main components:

(1) camphor > α-thujone > 1,8-cineol > β-thujone;
(2) camphor > α-thujone > β-thujone > 1,8-cineol;
(3) β-thujone > camphor > 1.8-cineol > α-thujone;
(4) 1,8-cineol > camphor > α-thujone > β-thujone;
(5) α-thujone > camphor > β-thujone > 1,8-cineneol [111].

#### 2.8.3. Antimicrobial Activity of Sage Oil

The use of sage EO in phytopharmacy has recently been described in two review articles [9,103]. Both the plant raw material as well as the extracts and EO obtained from sage are used as herbal medicines with mainly sanitization and antiseptic effects. EO of *Salvia officinalis* was active against severe acute respiratory coronavirus SARS-CoV (RNA virus), which was obtained from the sputum of a patient hospitalized with a diagnosis of SARS (severe acute respiratory syndrome) in Frankfurt University Hospital. It is worth noting that the overriding clinical feature of SARS is the rapidity with which many patients develop symptoms of acute respiratory distress syndrome (ARDS). Essential oil was weakly active at IC_50_ = 870 mg/ mL [112].

Sage EO has antibacterial activity against *Escherichia coli*, *Bacillus subtilis* [113], *Salmonella typhi*, *S. enteritidis*, *Shigella sonei* [114], *Staphylococcus aureus* [115], *S. epidermidis*, *S. mutans* [116], and *Shigella sonei* [9]. It is active against Gram-positive and Gram-negative bacteria [117]. The antimicrobial activity of the sage EO is attributed mainly to the presence of camphor, thujone, and 1,8-cineole [103].

Moreover, plants of the genus *Salvia* are used as a component of herbal teas and as flavoring for food. At the same time, the number of microorganisms developing tolerance to existing preservative techniques is constantly growing, and the unwillingness to use synthetic preservatives and antibiotics is prevalent. Therefore, the interest for food processors and consumers in antibacterial preservatives of plant origin, including the possibility of using EOs, is increasing [9]. For example, sage EO at a concentration of 2.0% has a bacteriostatic effect on the strains of *Salmonella anatu*m [118] and *Salmonella enteritidis* growing in minced meat. Unfortunately, such a high concentration of essential oil had a negative effect on taste. This problem was solved by using a lower concentration of the sage EO and the addition of sodium chloride, and the products were stored at a low temperature. However, with other meat products, there may be a problem with the formulation of the finished product, because both the NaCl concentration and the sage EO can negatively affect the taste sensation [119].

Because of the observed high vapor permeability of sage EO, it can be used as a disinfectant against airborne microorganisms, such as *Bacillus, Pseudomonas, Enterococcus hirae, Staphylococcus aureus, Aeromonas hydrophila, Aeromonas sobria*, and *Klebsiella oxytoca* [120,121]. Sage EO is also active against yeast *Candida albicans* [115]*, Candida glabrata, Candida krusei* and *Candida parapsilosis* [103] and fungal strains, such as *Aspergillus carbonarius* [121,122]*, Aspergillus niger* [123]*, Ashbiya gossypii, Rhizopus oryzae, Trichoderma reesei* [124]*, Alternaria solani, Ascochyta rabies, Botrytis cinerea, Monilia laxa, Penicllum italicum*, and *Rhizoctonia solani* [125].

### 2.9. Tea Tree EO

#### 2.9.1. Source

Tea tree EO is a product of water vapor distillation of leaves and top twigs of *Melaluleca alternifolia* Maiden and Betch and other *Melaleuca* species (*M. linariifolia* Smith, *M. dissitiflora* F. Mueller) [33]. According to research [126] aimed at standardizing the raw material for the production of essential oil for pharmaceutical purposes, *Melaleuca alterniforia* is a shrub with a height of about 7 m, characterized by stratified and flaky bark and oblong, pointed leaves with a characteristic pattern.

#### 2.9.2. Chemical Composition of Tea Tree EO

The main components of tea tree EO are terpine-4-ol (≥30%), teripene (about 20%), α-terpinene (about 8%), ρ-cymene (about 8%), α-pinene (about 3%), terpinolene (about 3%), and 1,8-cineol (≤15%) [127]. However, it should be noted that individual *Melaleuca* species have a very diverse content. As shown by Amri et al. [128], cultivars growing in Tunisia (*M. armillaris, M. acuminata, M. styphelioides*) may contain only traces of terpinene-4-ol, γ-terpinene, or ρ-cymene, in favor of *trans*-pinocarveol (25.1% *M. acuminata*), eugenol methyl (91.1% *M. styphelioides*), or *cis*-calamene (19% of *M. armillaris*). The analyses of more than 800 samples of tea tree EO has identified over 100 of its components.

#### 2.9.3. Antimicrobial Activity of Tea Tree EO

So far, the most promising tea tree EO in terms of antimicrobial properties is derived from a chemotype characterized by 30–40% terpinen-4-ol content [8]. EO was active against *Herpes simplex* virus type 1 in vitro. IC_50_ of this EO was determined as 2.0 µg/mL. EO was able to suppress viral multiplication by >96% [8,34]. Additionally, a randomized, placebo-controlled, investigator-blinded protocol was used to evaluate the efficacy of tea tree essential oil (6% tea tree EO gel) in the treatment of recurrent herpes labialis. The median time of reepithelization after treatment with this EO was 9 days compared to 12.5 days after placebo, indicating some benefit from tea tree EO treatment [129]. Meanwhile, the antibacterial activity has been determined to a much greater extent. The tea tree EO inhibited the growth of *S. aureus* and *E. coli* at a concentration of 0.78% and inhibited the adhesion of *S. mutans* [130] and the development of *Listeria monocytogenes* ATCC 7644 (MIC = 0.10 μL/g) in ground beef [131].

Tea tree EO, thanks to its antimicrobial properties, has been used in products for oral hygiene and dermatological applications [129,132]. Furthermore, Graziano et al. [126] determined that tea tree EO reduces the growth of bacteria responsible for halitosis—*Porphyromonas gingivalis* (MIC and MBC = 0.007%) and *Porphyromonas endodontalis* (MIC = 0.007% and MBC = 0.5%). The most important element of the study was the finding that tea tree EO not only inhibits the growth of bacteria responsible for halitosis leading to personal and social discomfort, but also limits the production of volatile sulfur compounds—H_2_S by *P. gingivalis* and CH_3_SH by *P. endodontalis*. Moreover, tea tree EO has shown an inhibitory effect against *Candida albicans*. For good therapeutic activity, the tea tree EO must contain terpineol at a minimum of 30% and a maximum of 15% cineol to simultaneously achieve very low skin irritation [129]. The aim of the study conducted by Mertas et al. [131] was the reduction of yeast resistance to the drug fluconazole due to the simultaneous use of tea tree EO or its main component (terpinen-4-ol). The authors indicated that as many as 62.5% of the 32 resistant strains became susceptible to the drug after 24 h exposure to the essential oil or its component. The minimum fluconazole concentration inhibiting fungal growth also decreased significantly (from 244.0 μg/mL to 38.46 μg/mL), along with its minimum fungicidal concentration (from 254.47 μg/mL to 66.62 μg/mL).

Recently [8], the antimicrobial properties of commercially available tea tree EOs were investigated. Of the 10 EOs, 5 were active. Identified components of the tea tree EO reduced the survival of the bacteria in the *Pseudomonas aeruginosa* biofilm and caused oxidative damage in *Candida glabrata*. On the other hand, EO of *Melaleuca alternifolia*) had only marginal antifungal activity against *Aspergillus niger* (MIC = 625 µg/mL), attributed to its active components terpinen-4-ol and α-terpineol [133]. Another example is *Penicillium expansum,* which is a cosmopolitan pathogen that can cause damage to fruit crops. Germination of this fungus was completely inhibited at the concentration of tea tree EO 250 µg/mL. Tea tree EO caused damage to the plasma membrane, leading to DNA, protein, lipid, and glucose leakage [134]. In turn, the effects of tea tree EO on mitochondrial morphology and function in a culture of *Botrytis cinerea* was investigated. This EO at a concentration of 2 mL/L severely damaged mitochondria, resulting in matrix loss and increased mitochondrial irregularity. The next consequence of mitochondrial damage was the disruption of the tricarboxylic acid cycle and the accumulation of reactive oxygen species [135].

## 3. Conclusions

Contrary to common opinion, limited EOs possess demonstrated potential as antimicrobial agents. It should be emphasized that although the antimicrobial activity is well established, the real effect is significantly weaker compared to synthetic compounds (including antibiotics). Gram-positive bacteria seem to be much more susceptible to essential oil than Gram-negative organisms. According to available data, the activity is usually correlated with phenolic, aromatic, or alcohol groups. Due to their high volatility, the effective time of action is limited, and features such as encapsulation could be changed. On the other hand, low toxicity level, as well as their natural origin, makes them an attractive alternative in both the food as well as in cosmetic industries. Several practical application could be implemented in these industries. In summary, it should be underlined that the use of EO in microbial stabilization is possible, but all cases must be individually examined.

## Supplementary Materials

The following are available online. Figure S1. The chromatogram of lavender oil; Figure S2. The chromatogram of thyme oil; Figure S3. The chromatogram of peppermint oil; Figure S4. The chromatogram of cajeput oil; Figure S5. The chromatogram of cinnamon oil; Figure S6. The chromatogram of eucalyptus oil; Figure S7. The chromatogram of clove oil; Figure S8. The chromatogram of sage oil; Figure S9. The chromatogram of tea tree oil; Table S1. Chemical composition of Eos; Table S2: The antimicrobiological activity of EOs, Materials and Methods.
