# Supplementary material for: Essential Oils as Antimicrobial Agents—Myth or Real Alternative?
Source: Molecules. 2019 Jun 5;24(11):2130. doi: 10.3390/molecules24112130 (PMC6612361; doi:10.3390/molecules24112130)
Supplement: Supplementary file 1 [file molecules-24-02130-s001.pdf]

Supplementary data

Essential Oils as Antimicrobial Agents—Myth or Real Alternative?

Katarzyna Wińska, Wanda Mączka, Jacek Łyczko, Małgorzata Grabarczyk, Anna Czubaszek, Antoni Szumny

Samples and materials:

Samples of essential oil were obtained from local distillery (Herbiness, Poland).

GC-MS Analysis

GC-MS analysis was performed on Shimadzu GCMS-QP2020 NX apparatus (Shimadzu, Kioto, Japan) equipped with a ZB-5 (30 m x 0.25 mm x 1.40 µm) column Phenomenex (US). Briefly, the GC oven temperature was programmed from 50 °C to 130°C at a rate of 4.0 °C and holding for 2 min, then to 280°C at a rate of 10.0°C and holding for 5 min. Samples were injected at 1:10 split ratio and helium was used as a carrier gas with flow rate of 1.0 ml/min. Scanning was performed from 35 to 500 m/z with an absolute detector voltage of 1 kV. Ionization was performed in electron impact (EI) mode at 70 eV.

Identification and quantification of Volatile Compounds

Identification of volatile compounds was based on the comparison of experimentally obtained mass spectra with mass spectra available in NIST 14 database. Moreover, obtained retention indices (RI) by Kovats were compared with RI available in literature sources. The data proceeding was performed using GCMS Postrun Analysis software (Shimadzu, Kioto, Japan). Quantification was based on internal standard (2-undecanone), as well as *n*-hexanol calibration curves.

Figure S1. The chromatogram of lavender oil.

Figure S2. The chromatogram of thyme oil.

Figure S3. The chromatogram of peppermint oil.

Figure S4. The chromatogram of cajeput oil.

Figure S5. The chromatogram of cinnamon oil.

Figure S6. The chromatogram of eucalyptus oil.

Figure S7. The chromatogram of clove oil.

Figure S8. The chromatogram of sage oil.

Figure S9. The chromatogram of tea tree oil.

Table S1. Chemical composition of EOs.

Table S2. The antimicrobiological activity of EOs.

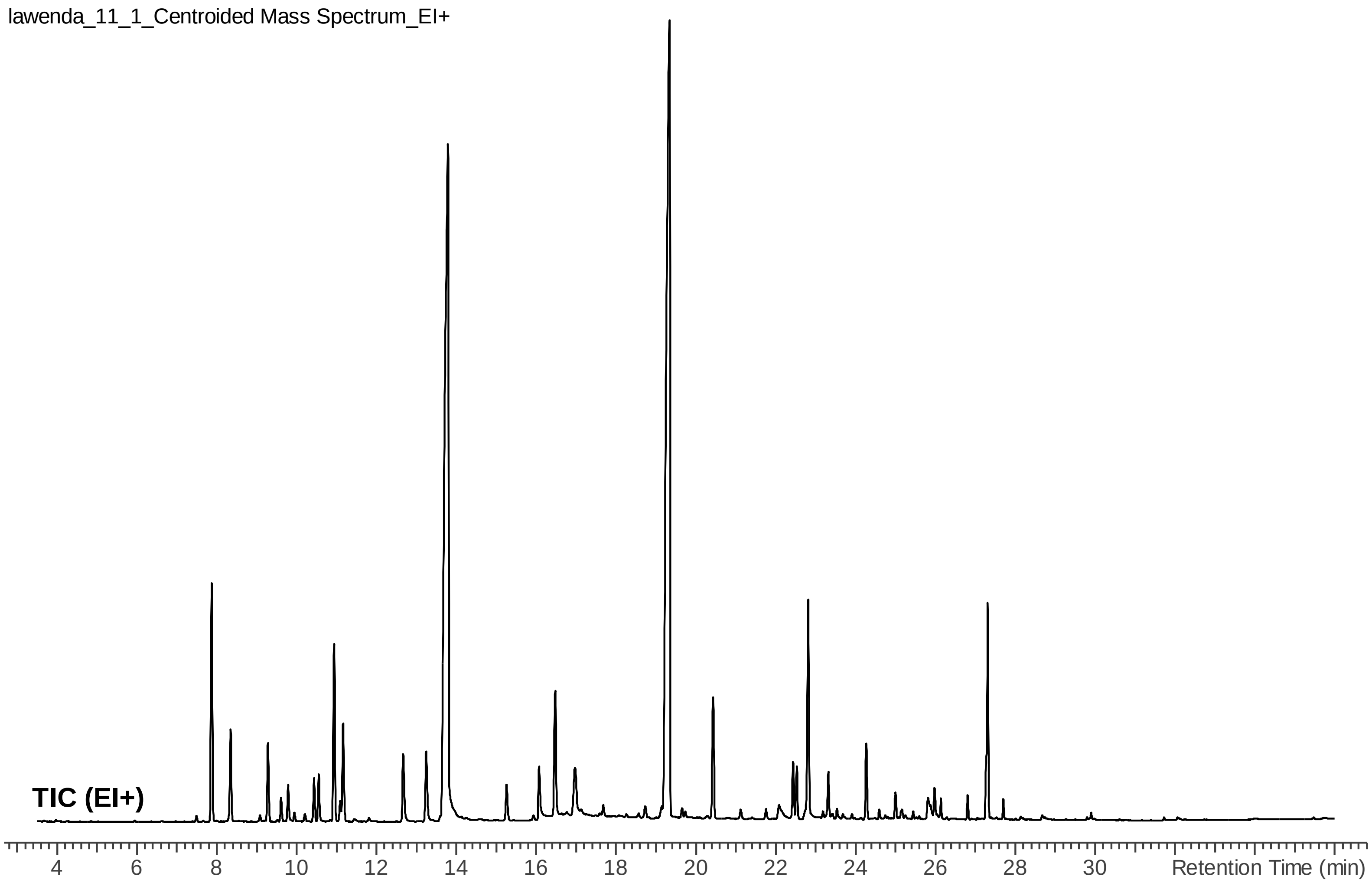

Figure S1. The chromatogram of lavender oil.

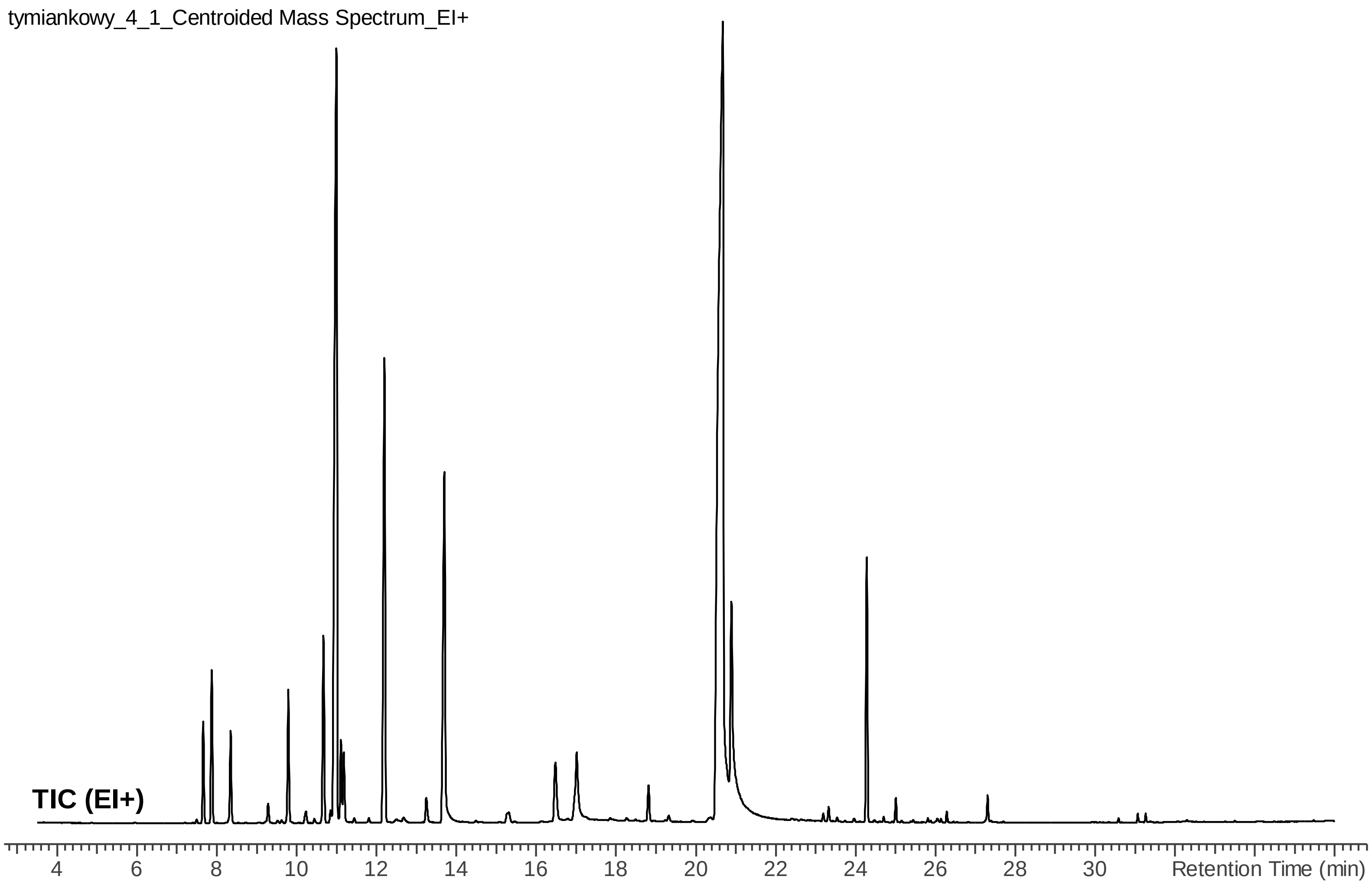

Figure S2. The chromatogram of thyme oil.

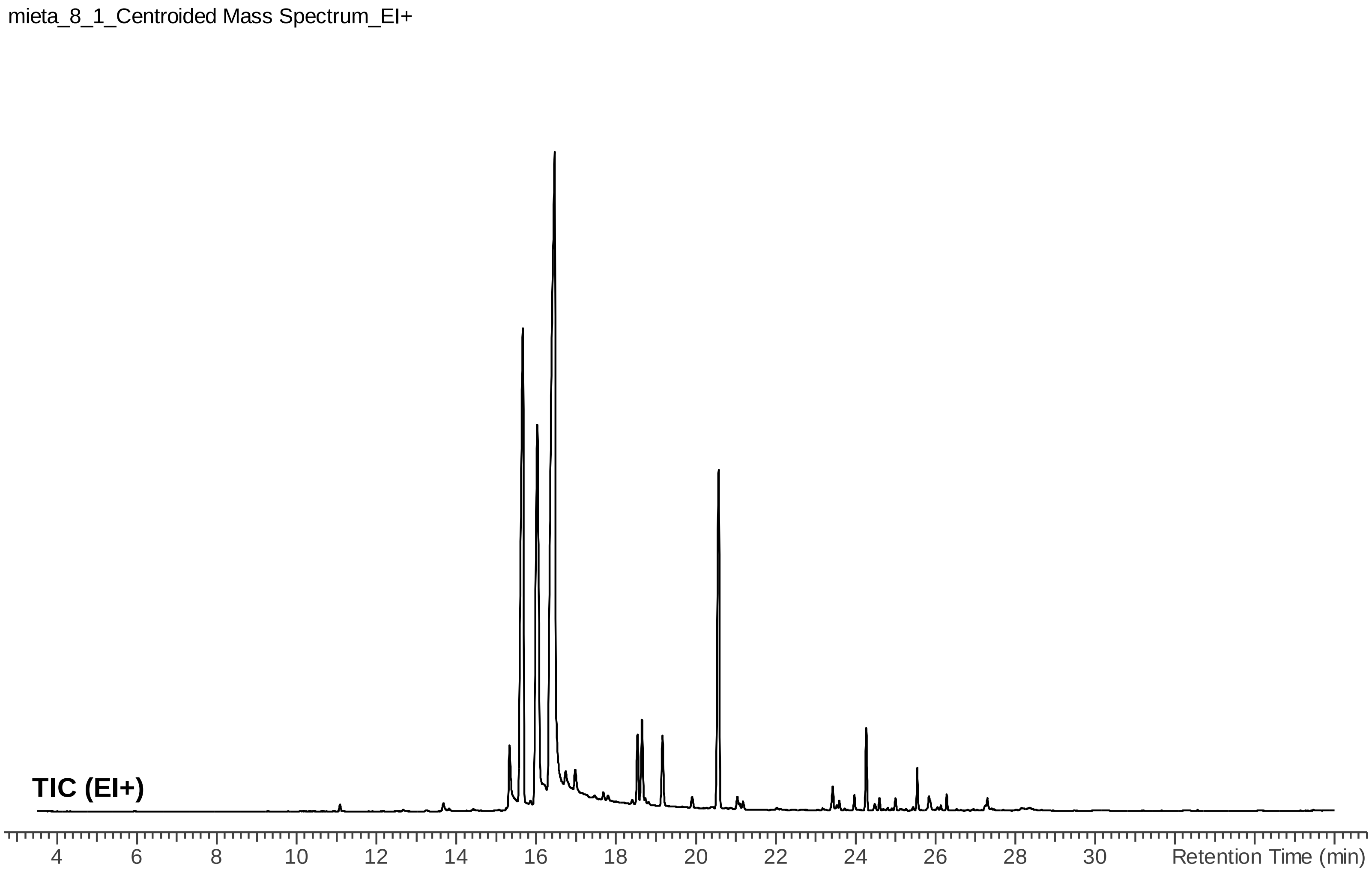

Figure S3. The chromatogram of peppermint oil.

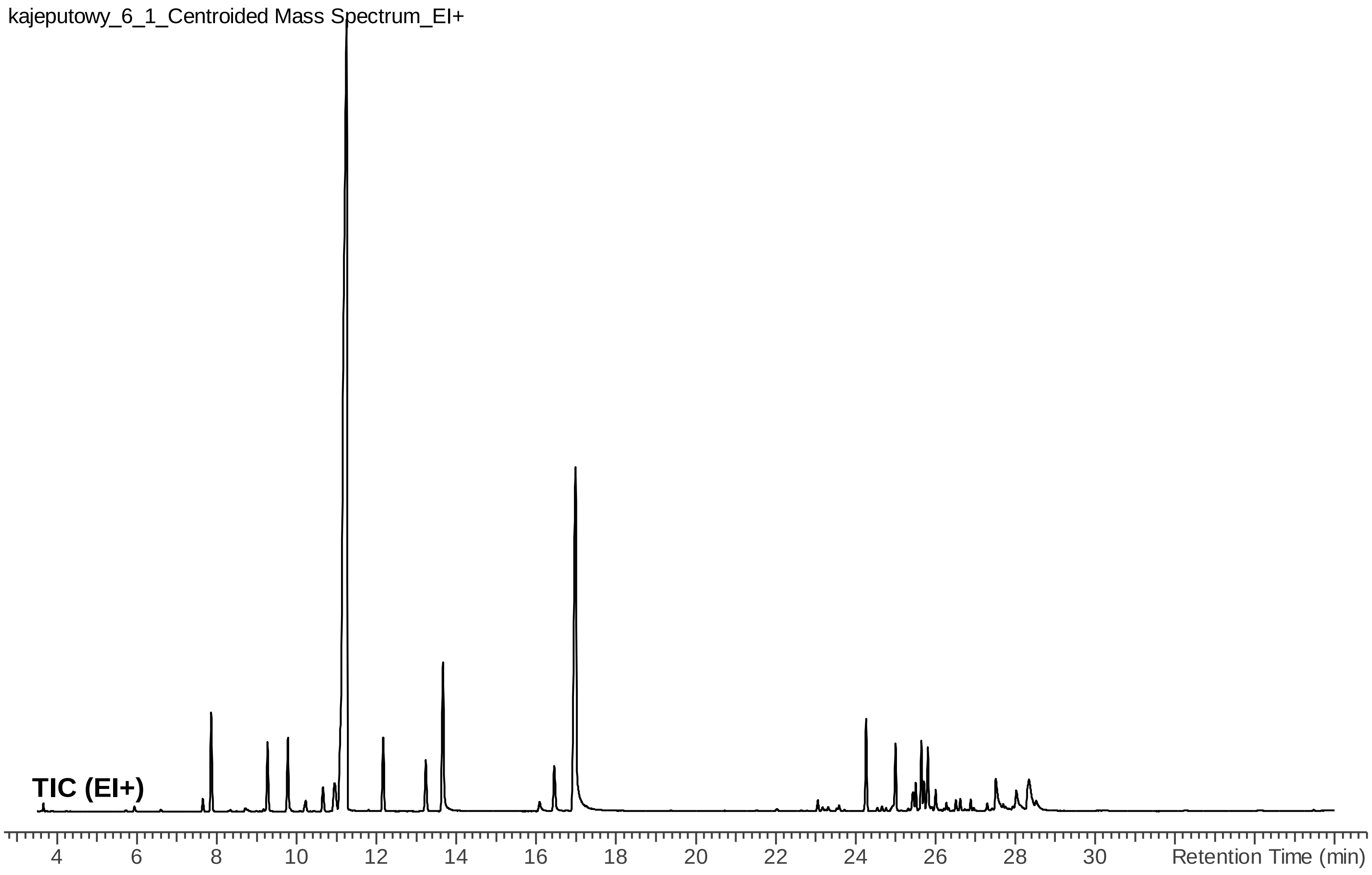

Figure S4. The chromatogram of cajeput oil.

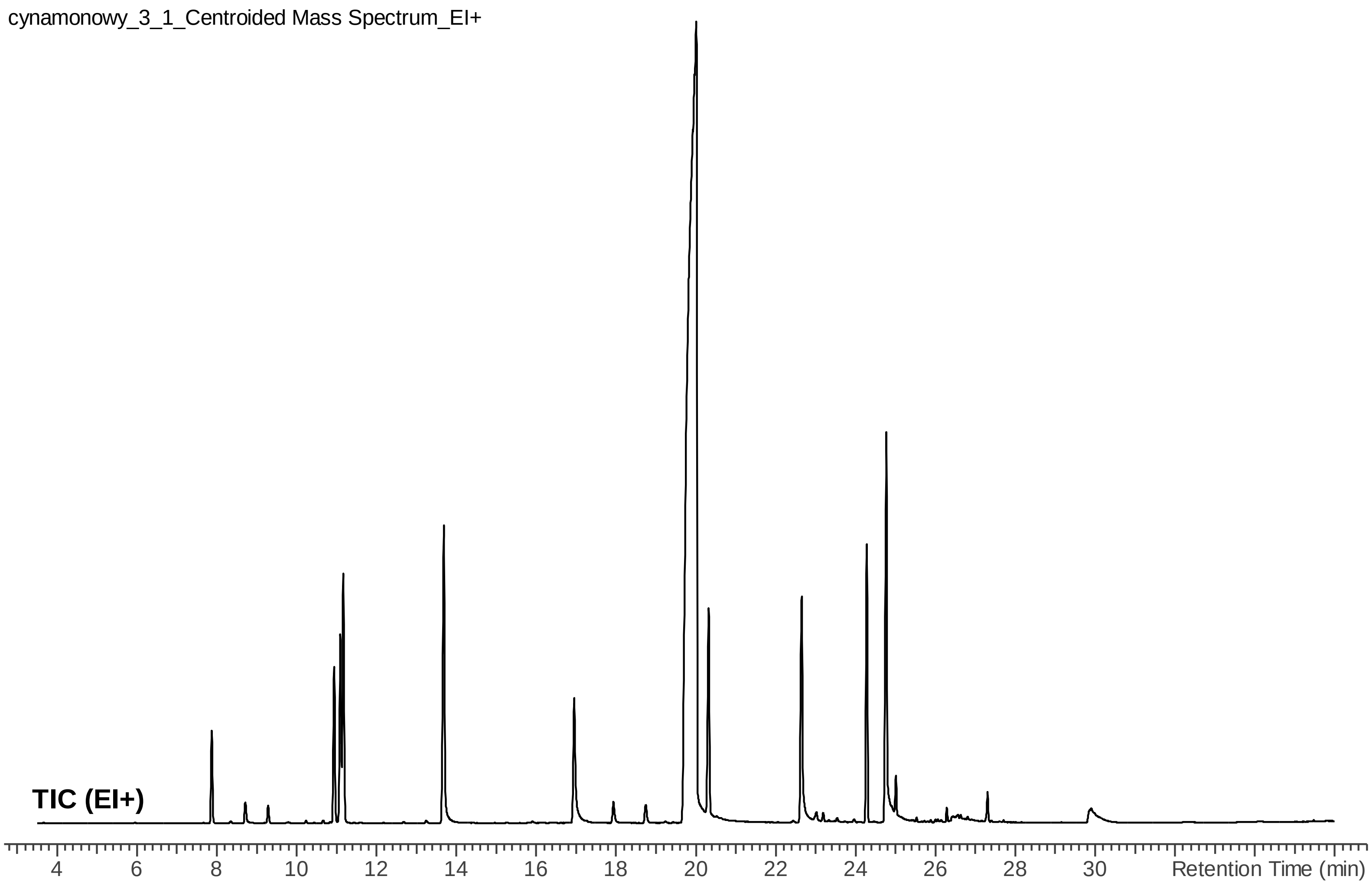

Figure S5. The chromatogram of cinnamon oil.

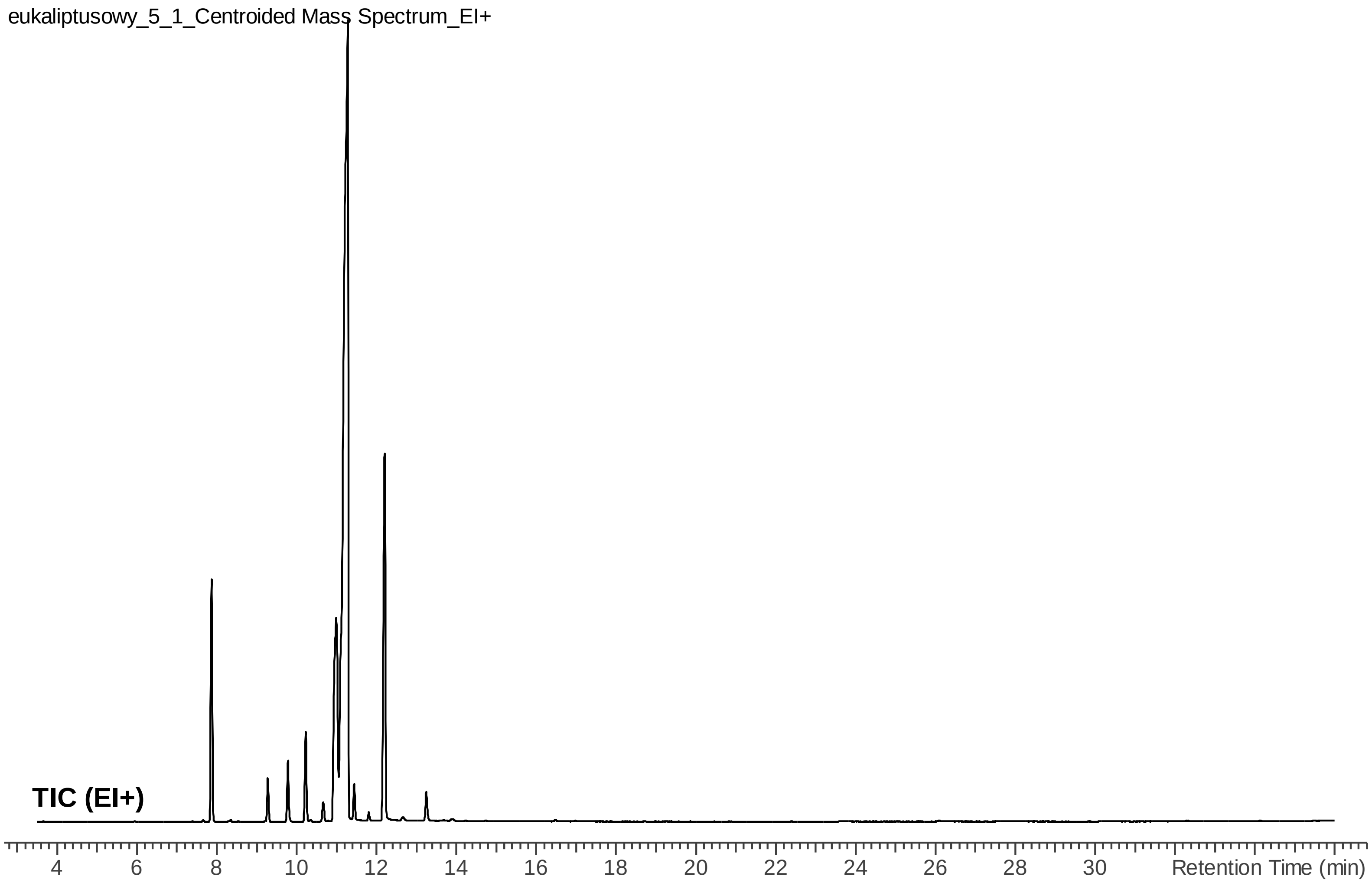

Figure S6. The chromatogram of eucalyptus oil.

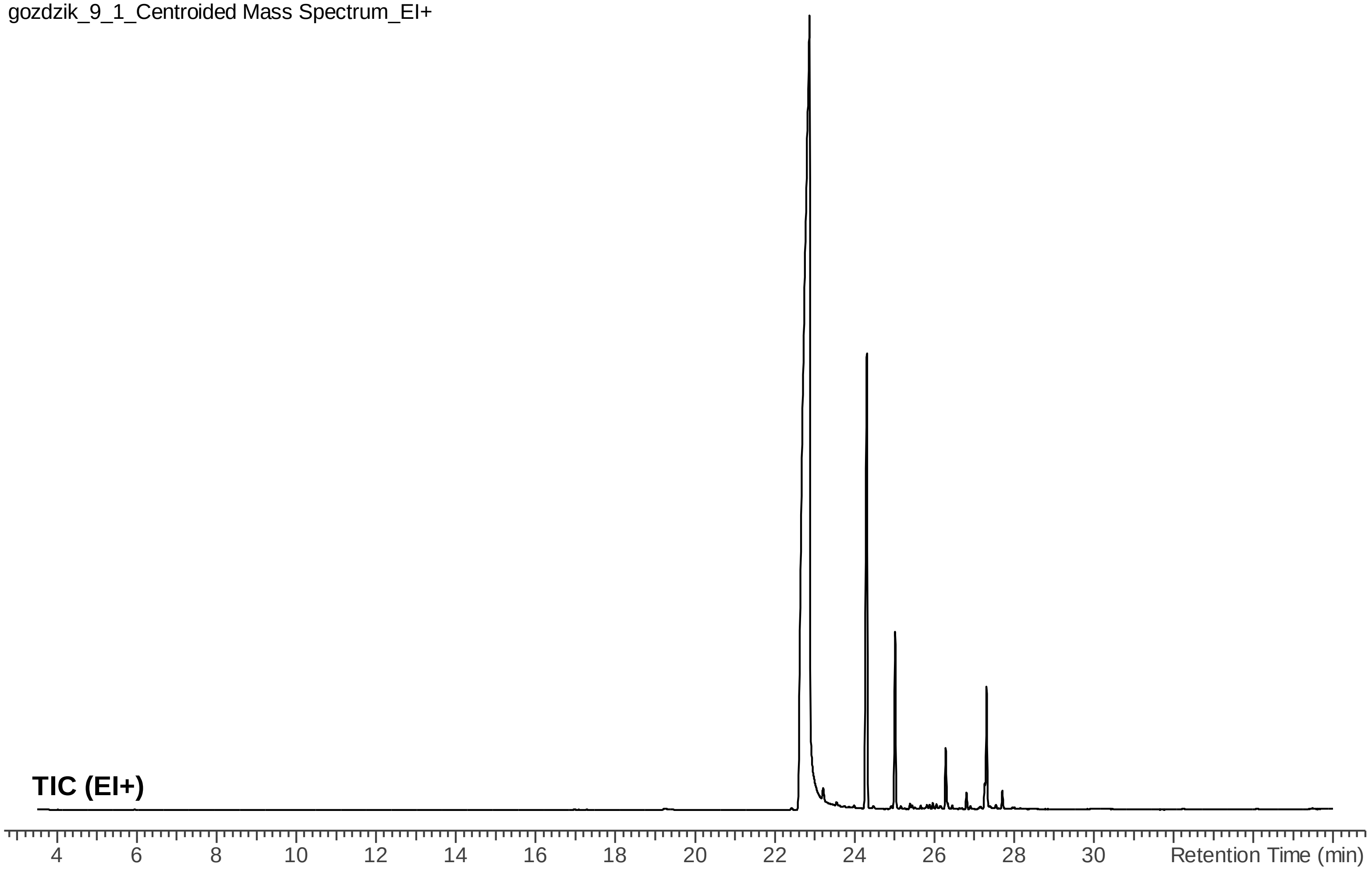

Figure S7. The chromatogram of clove oil.

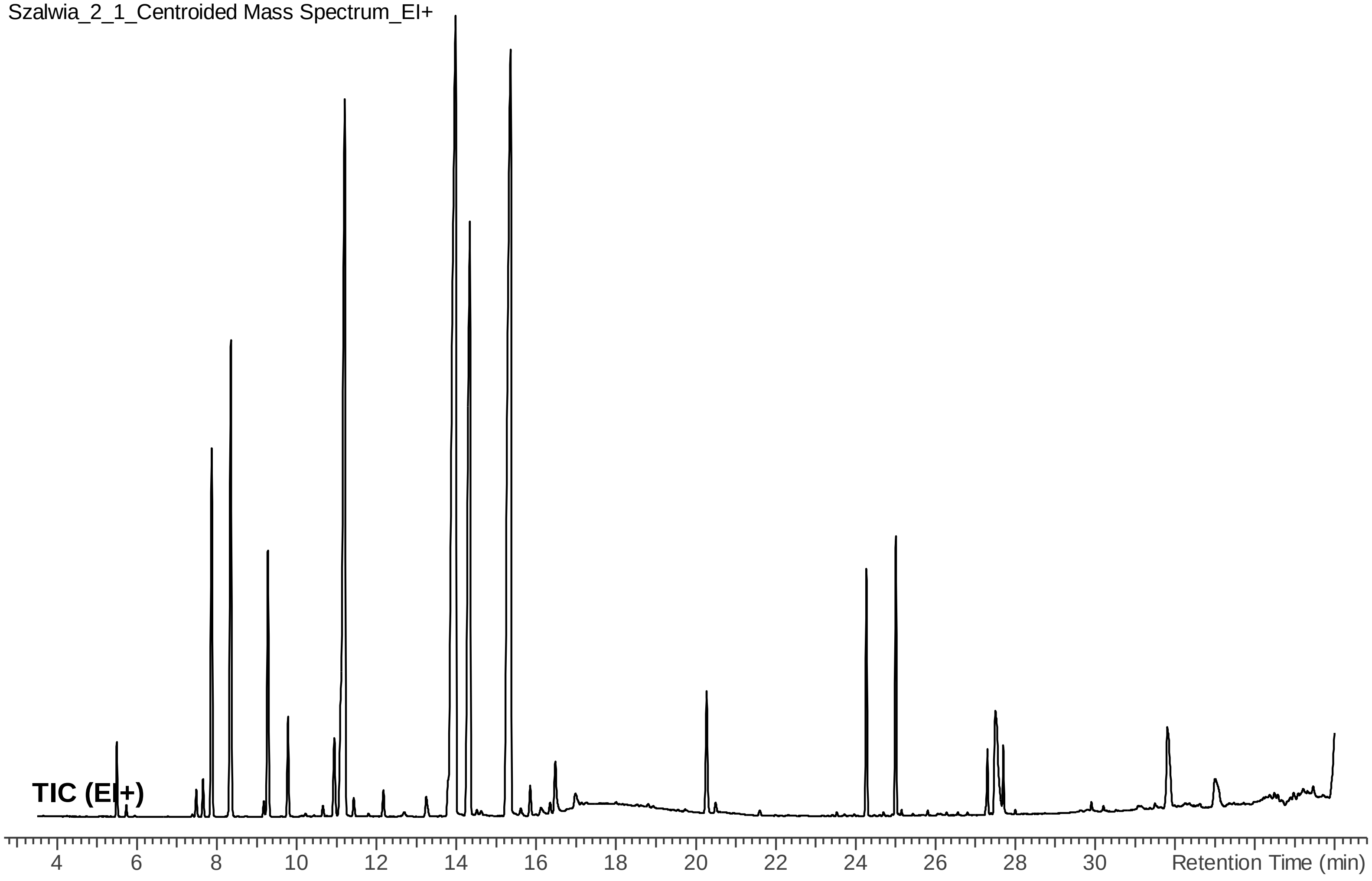

Figure S8. The chromatogram of sage oil.

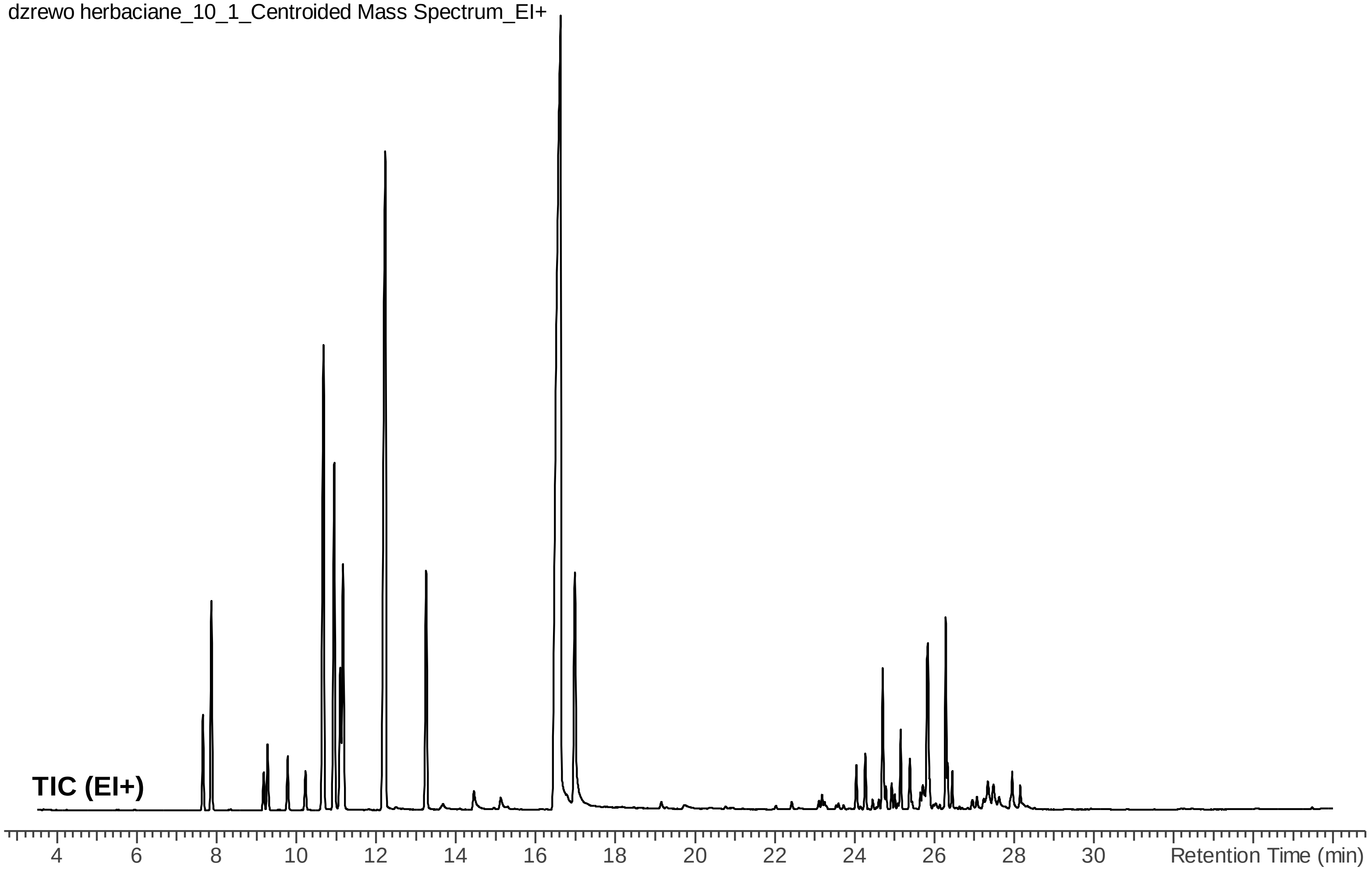

Figure S9. The chromatogram of tea tree oil.

Table S1. Chemical composition of EOs.

	Rt	**Lavender oil**	**Thyme oil**	**Peppermint oil**	**Cajeput oil**	**Cinnamon oil**	**Clove oil**	**Eucalyptus oil**	**Sage oil**	**Tea tree oil**
Z-Salvene	5.48								0.63	
E-Salvene	5.73								0.10	
β-Picoline	5.937				0.13			0.01		
Tricyclene	7.45								0.27	
α–Thujene	7.656		1.26		0.29			0.04		1.17
α-Pinene	7.868	3.71	2.07		2.39	1.18		6.24	4.22	2.80
α-Fenchene	8.285		1.17					0.02	5.85	
Camphene	8.343							0.04		
(2*E*)-Heptenal	8.343	1.42				0.02				
Thuja-2,4(10)-diene	8.531							0.01		
Benzaldehyde	8.713					0.31				
Sabinene	9.179								0.13	0.50
β-Pinene	9.277	1.27	0.24		1.76	0.23		1.08	3.10	0.90
Octan-3-one	9.608	0.36								
Myrcene	9.781	0.61	1.79		1.87			1.58	1.07	0.70
α-Phellandrene	10.227		0.21		0.34	0.03		2.44	0.03	0.53
Linalool oxide <dehydro-, cis->	10.345							0.05		
delta-3-Carene	10.435	0.69								
Hexyl acetate	10.552	0.73								
α-Terpinene	10.681		2.68		0.66	0.03		0.58	0.12	7.89
para-Menth-1-ene	10.850		0.18							
Cymene <para->	10.948	2.99	18.50		1.22	2.22		12.17	1.03	5.38

Limonene	11.089	0.34	1.15	0.13		2.79			14.15	
Sylvestrene	11.102									2.10
Eucalyptol	11.171	1.66	0.97		50.18	3.48		61.29		3.65
(Z)-β-Ocimene	11.440							0.89	0.20	
(E)-β-Ocimene	11.806							0.21	0.03	
γ-Terpinene	12.230		8.14		2.01			12.22	0.30	14.70
cis-Linalool oxide	12.670	1.28						0.13	0.06	
cis-Linalool oxide	13.244	1.37								
para-Cymenene	13.25								0.31	
Terpinolene	13.256		0.42		1.47			0.84		3.76
Linalool	13.673	28.45	7.14	0.21	4.61	5.15			23.30	0.12
Solusterol	13.870							0.04		
Hex-2-enal <2-isopropyl-, 5-methyl->	13.916							0.05		
β-Thujone	14.3								11.63	
cis- para-Menth-2-en-1-ol	14.452									0.43
α-Campholenal	14.51								0.05	
allo-Ocim-(4E,6Z)-ene	14.728							0.01		
trans-para-Menth-2-en-1ol	15.125									0.23
Camphor	15.258	0.68	0.31						20.32	
Isopulegol	15.335			2.34						
Menthone	15.667			18.20					0.09	
trans-Pinocamphone	15.857			0.15					0.36	
Menthone	16.027			15.69						
Borneol	16.071	0.96								
δ-Terpineol	16.085				0.32				0.15	
cis-Pinocamphone	16.35								0.12	
Menthol	16.461			40.24						
*Iso*Menthol	16.736			0.42						

Terpinen-4-ol	16.623	2.42				2.58		0.05	0.80	33.94
Terpineol <alpha->	16.984	1.38	1.84	0.55	15.41				0.28	4.65
n-Decanal	17.457			0.07						
n-octyl acetate	17.680	0.16								
trans-Oct-2-enyl acetate	17.797			0.11						
(Z)-Cinnamaldehyde	17.933					0.37				
3-Hexenyl 2-methylbutyrate	18.407			0.08						
cis-3-hexenyl-Isovalerate	18.539			1.54						
Pulegone	18.652			2.08						
hexyl-, 3-methyl butanoate < ->	18.735	0.21		0.13						
o-Anisaldehyde	18.742					0.35				
Carvacryl methyl ether	18.812		0.56							
Carvone	18.820			0.06						
Carvotanacetone	19.163			1.69						
cis-Piperitone oxide <	19.140	0.22								
Dec-(4Z)-en-1-ol	19.153									0.11
Neobergamate	19.336	2.19	0.07							
Neomenthyl acetate	19.906			0.24						
(*E*)-Cinnamaldehyde	20.007					61.48				
isobornyl acetate	20.26								1.60	
(*E*)-Anethole	20.319					3.48				
Lavandulyl acetate	20.433	31.09								
Bornyl acetate	20.49								0.13	
Menthyl acetate	20.568			9.39						
Thymol	20.674		39.14							
Carvacrol	20.890		6.03							
Isomenthyl acetate	21.038			0.29						
Nonyl acetate	21.100			0.12						

cis- -α Terpinyl acetate	21.124	0.18								
iso-Verbanol acetate	21.181			0.18						
Myrtenyl acetate	21.59								0.06	
n-hexyl-Tiglate	21.758	0.19								
Citronellyl acetate	22.087	0.64								
α-Cubebene	22.424									0.11
neoiso-Dihydro carveol acetate	22.438	1.03								
Carbonic acid <4-cycloocten-1-yl-, methyl-> ester	22.529	0.84								
Linalool isobutanoate	22.815	4.27								
Eugenol	22.649					4.08	78.84			
Propan-2-one <methoxy-3-phenyl->	23.019					0.15				
Longicyclene	23.055				0.10					
Isoledene	23.105									0.12
α–Copaene	23.185	0.23			0.27	0.12	0.25			0.20
2-epi-a- Funebrene	23.189		0.10							
β-Patchoulene	23.248									0.13
trans-Geranyl acetate	23.320	0.81			0.12					
But-(2E)-en-1-one <1-(2,4,4-trimethyl-, 2-cyclohexen-1-yl)->	23.325		0.20							
β Cubebene	23.538	0.18								
Tetradec-1-ene	23.548					0.04	0.06			
β-Elemene	23.596			0.52	0.13					0.08
Longifolene	23.957					0.05				
Decyl acetate	23.969			0.27						
α-Gurjunene	24.042									0.59
(*E*)-β–Caryophyllene	24.268	1.18	3.57	1.55	2.22	3.61	11.93		2.48	0.71

β-Copaene	24.479			0.15						
γ-Maaliene	24.452									0.12
α–Guaiene	24.545				0.07					
n-octyl-Isovalerate	24.598			0.20						
Maaliene <alpha->	24.609									0.12
Himachalene <alpha->	24.703		0.07						0.04	1.99
(*E*)-Cinnamyl acetate	24.770					6.73				
Selina-5,11-diene	24.786									0.26
trans-Muurola-3,5-diene	24.929									0.30
trans-Prenyl limonene	24.999		0.29		1.74					
α-Humulene	25.003	0.39		0.22		0.58	3.62		2.78	0.17
9-epi-(*E*)-Caryophyllene	25.154						0.06			0.95
trans-Dodec-2-en-1-ol	25.165	0.17								
Cadina-1(6),4-diene	25.385						0.10			0.62
γ-Muurolene	25.440			0.05						0.07
γ-Gurjunene	25.440				0.64					
Germacrene D	25.450			0.71			0.06			
α-Amorphene	25.507				0.59					
α-Curcumene	25.526					0.04				
β-Selinene	25.650				1.58					0.19
δ-Selinene	25.710				0.60					0.38
γ-Amorphene	25.755									0.18
α-Selinene	25.814				1.62		0.06			
1-Methyl-3-(4-methylpent-3-enyl)cyclohex-3-ene-1-carboxaldehyde	25.815	0.78								
Bicyclogermacrene	25.836			0.37						3.33
α-Muurolene	25.883						0.06			
β-Dihydroagarofuran	25.910				0.09					

(*E,E*)-a-Farnesene	25.960						0.10			
Lavandulyl <2-methyl-> butyrate	25.979	0.60								
β-Bisabolene	25.995					0.02				
δ-Amorphene	26.005				0.52					
δ-Cadinene	26.135			0.08						
α-Bulnesene	26.035									0.12
γ–Cadinene	26.137	0.28								
Z-nerolidol	26.286		0.11	0.25	0.17	0.15	1.20			2.12
γ-Cuprenene	26.334									0.44
Cadina-1,4-diene <trans->	26.448						0.06			0.41
Selina-4(15),7(11)-diene	26.510				0.26					
Selina-3,7(11)-diene	26.622				0.23					
α-Calacorene	26.631					0.03				
epi-Longipinanol <	26.808	0.30				0.03	0.30			
Longipinanol	26.883				0.21					
Guaiol	26.954									0.16
Ledol	27.068									0.18
Fokienol	27.236			0.11						0.15
Caryophyllene oxide	27.311	3.50		0.22	0.16	0.37	2.69		0.63	
Viridiflorol	27.339		0.31						2.62	0.67
Guaiol	27.480									0.52
Cubenol <1,10-di-epi->	27.512				1.37					
Eremoligenol	27.623									0.21
allo-Aromandendrene epoxide	27.7								0.48	
Bulnesol	27.951									0.62
Patchoulyl acetate	28.027				0.62					
Caryophyllene acetate	28.154									0.22
Guaiac acetate	28.341				2.00					

Guaiac acetate	28.520				0.41					
β-Bisabolenol	29.904					0.30				
Manoool	30.0								0.08	
(5Z,9E)-Farnesyl acetone	31.067		0.09							

Table S2. The antimicrobiological activity of EOs.

Essential oil	Main constituents	Microorganism	Reference
Lavandula angustifolia Mill.	linalyl acetate<br>linalool	*Herpes simplex*	[15]
		Staphylococcus aureus (MRSA)	[17]
		S. aureus ATCC 25923	[13]
		S. aureus MRSA / ORSA	
		Escherichia coli [enro (-)]	
		Pseudomonas aeruginosa	
		Salmonella typhimurium ATCC 14028	
		Candida albicans ATCC 10231	
		Enterococcus sp. (VRE)	[18]
		Shigella flexneri	[20]
		Haemophilus influenzae	[23]
		Aureobasidium pullulans	[25]
		Penicillium citrinum	
		Penicillium simplicissimum	
		Botrytis cinerea	[26]
		Fusarium solani var. *coeruleum*	
		Aspergillus niger	[27]
		Aspergillus tubingensis	
Thymus vulgaris L.	thymol<br>p-cymene<br>γ-terpinene	*Herpes simplex*	[32]
		influenza virus A1/Denver/1/57 (H1N1)	[33]
		Streptococcus mutans	[38]
		Streptococcus pyogenes	[39]

		S. aureus ATCC 25923	[28]
		K. pneumoniae ATCC 13882	
		Brachyspira hyodysenteriae	
		Malassezia furfur (drożdże)	[40]
		Fusarium graminearum Fg 06-17	[41]
		Aspergillus flavus	[43]
		Aspergillus niger	
		Botrytis cinerea ATCC12481	[44]
		Penicillium expansum	
Mentha piperita (L.) Hudson	menthol<br>menthone<br>cineol<br>menthyl acetate<br>isomenthone	*Herpes simplex*	[47]
		Escherichia coli WDCM 00013	[49]
		Listeria monocytogenes WDCM 00020	
		Pseudomonas aeruginosa WDCM 00024	
		Salmonella enterica WDCM 00030	
		Staphylococcus aureus WDCM 00032	
		Candida albicans	[7, 50, 52]
		Candida tropicalis	[50]
		Pichia anomala	
		Saccharomyces cerevisiae	
		Streptococcus mutans	[56]
		Alternaria alternata	[57]
		Aspergillus flavus	
		Aspergillus niger	
		Colletotrichum gloeosporioides	
		Fusarium solani	
		Macrophomina phaseol	
Melaleuca leucadendron L.	1,8-cyneole<br>γ-terpinene<br>α-pinene<br>viridiflorol	*Bacillus cereus*	[63]
		Bacillus subtilis	
		Corynebacterium diphtheriae	
		Corynebacterium minutissimus	
		Enterococcus faecium	
		Listeria monocytogenes	

		Staphylococcus epidermidis	
		Alcaligenes ,faecalis ATCC 356551	[65]
		Bacillus cereus ATCC 14579	
		Enterobacter cloacae ATCC 23355	
		Escherichia coli ATCC 25922	
		Streptococcus faecalis ATCC 19433	
		Staphylococcus aureus ATCC 259231	
		Micrococcus luteus ATCC 4698	
		Candida albicans ATCC 14053	
		Acinetobacter spp.	[59]
		Klebsiella spp.	
		Pseudomonas aeruginosa	
		Aspergillus niger	
Cinnamomum zeylanicum	*trans*-cinnamaldehyde<br>cinnamyl aldehyde<br>benzaldehyde<br>borneol<br>eugenol<br>cinnamic acid	HSV1	[72]
		H1N1	[33, 72]
		Acinobacter baumannii	[73]
		Staphylococcus aureus	[73, 77]
		Escherichia coli	[73, 77, 81]
		Pseudomonas aeruginosa	[73,77]
		Borrelia burgdorferi	[74]
		Enterococcus faecalis	[77]
		Salmonella typhimurium	[6]
		Listeria monocytogenes	
		Escherichia coli	[81]
		Candida albicans	
		Escherichia coli PTCC 1163	[79]
		Staphylococcus aureus PTTC 25923	
		Aspergillus niger	[83]
		Cladosporium herbarum	
		Fusarium culmorum	
		Fusarium oxysporum	
		Fusarium solani	

		Fusarium verticillioides	
		Fusarium poae	
Cinnamomum cassia (L.) J. Presl		*Streptococcus mutans* ATCC 35668	[80]
Eugenia caryophyllata Thunb	eugenol<br>eugenol acetate<br>anethol<br>benzyl salicylate<br>β-caryophyllene	*Herpes simplex*	[32, 55, 87]
		Bacillus cereus WU10	[89]
		Escherichia coli WU40, W1485, K12	
		Listeria innocua WU 507	
		Salmonella typhimurium WU73	
		Aeromonas hydrophila ATCC 7966	[90]
		Candida albicans ATCC 10231	
		Klebsiella pneumoniae ATCC 13883	
		Proteus mirabilis ATCC 10005	
		Pseudomonas aeruginosa ATCC 27853	
		Staphylococcus aureus ATCC 6538,	
		Staphylococcus epidermidis ATCC 14990	
		Escherichia coli ATCC 8739	
		Streptococcus pyogenes	[10]
		Campylobacter jejuni	
		Haemophilus influenzae	
		Streptococcus agalactiae	
		Staphylococcus aureus	
		Klebsiella pneumoniae	
		Salmonella enteritidis	
		Bacilus subtilis	
		Morganella morganii	
		Yersinia enterocolitica	
		Listeria monocytogenes	[10, 92]
		Candida albicans	[91]
		Aspergillus niger	[86]
		Penicillum christopherum	
		Mycobacterium phlei	
Eucalyptus globulus Labill	1,8-cineol	*Herpes simplex*	[32]

	limonene<br>α-pinene<br>γ-terpinene<br>α-terpineol	mumps virus	[100]
		Coxsakievirus B3 Nancy	[9,98]
		Acinetobacter baumannii	[101]
		Porphyromonas gingivalis	
		Streptococcus mutans	
		Edwardsiella tarda FP 5060	[102]
		Lactococcus garviae FP 5245	
		Photobacterium damselae FP 4101	
		Streptococcus iniae FP 5228	
		Streptococcus parauberis FP 3287	
		Vibrio harveyi FP 8370	
		Vibrio ichthyoenteri FP 4004	
		Staphylococcus aureus	[103]
		Escherichia coli	
		Aureobasidium pullulans L6F	[95]
		Candida diversa T6D	
		Hansenula polymorpha CBS 4732	
		Pichia fermentans T2A1	
		Pichia kluyveri T1A	
		Pichia anomala	
		Saccharomyces cerevisiae SPA	
		Zygosaccharomyces bailii	
		Alternaria alternata	[104]
		Fusarium roseum	
		Mucor hiemalis	
		Penicillium glabrum	
Salvia officinalis L.	tamphor<br>α-thujone<br>1,8-cineol<br>β-thujone;	coronavirus SARS-CoV	[114]
		Aeromonas hydrophila,	[122]
		Klebsiella oxytoca	
		Aeromonas sobria	
		Bacillus megatherium	
		Bacillus cereus	[9, 122]

		Bacillus subtilis	[9, 115, 122]
		Escherichia coli,	[115]
		Salmonella anatum	[120]
		Salmonella typhi	[9, 116]
		Shigella sonei	
		Salmonella enteritidis	[116]
		Staphylococcus aureus	[9, 117]
		Candida albicans	
		Staphylococcus epidermidis	[118]
		Streptococcus mutans	
		Candida glabrata	[105]
		Candida krusei	
		Candida parapsilosis	
		Aspergillus carbonarius	[123, 124]
		Aspergillus niger	[9, 125]
		Ashbiya gossypii	[126]
		Trichoderma reesei	[126]
		Rhizopus oryzae	[126]
		Botrytis cinerea	[127]
		Alternaria solani	
		Ascochyta rabies	
		Monilia laxa	
		Penicllum italicum	
		Rhizoctonia solani	
Melaluleca alternifoli<br>*Tea tree*<br>*Tea tree* cd	terpine-4-ol<br>teripene<br>α-terpinene<br>ϱ-cymene	*Herpes simplex* virus type 1 (HSV-1)	[8, 32, 131]
		Escherichia coli	[132]
		Staphylococcus aureus	
		Streptococcus mutans	
		Porphyromonas endodontalis	[129]
		Porphyromonas gingivalis	
		Candida albicans	[132, 134]
		Candida glabrata	[8]

		Aspergillus niger	[135]
		Penicillium expansum	[136]
		Botrytis cinerea	[137]
